# The diversity of reproductive parasites among arthropods: *Wolbachia *do not walk alone

**DOI:** 10.1186/1741-7007-6-27

**Published:** 2008-06-24

**Authors:** Olivier Duron, Didier Bouchon, Sébastien Boutin, Lawrence Bellamy, Liqin Zhou, Jan Engelstädter, Gregory D Hurst

**Affiliations:** 1 University College London, Department of Biology, Stephenson Way, London NW1 2HE, UK; 2 Université de Poitiers, Ecologie Evolution Symbiose, UMR CNRS 6556, Avenue du Recteur Pineau, 86022 Poitiers, France; 3 Institute of Integrative Biology (IBZ), ETH Zurich, Universitätsstrasse, ETH Zentrum, CHN K12.1, CH-8092 Zurich, Switzerland; 4 University of Liverpool, School of Biological Sciences, Crown Street, Liverpool L69 7ZB, UK

## Abstract

**Background:**

Inherited bacteria have come to be recognised as important components of arthropod biology. In addition to mutualistic symbioses, a range of other inherited bacteria are known to act either as reproductive parasites or as secondary symbionts. Whilst the incidence of the α-proteobacterium *Wolbachia *is relatively well established, the current knowledge of other inherited bacteria is much weaker. Here, we tested 136 arthropod species for a range of inherited bacteria known to demonstrate reproductive parasitism, sampling each species more intensively than in past surveys.

**Results:**

The inclusion of inherited bacteria other than *Wolbachia *increased the number of infections recorded in our sample from 33 to 57, and the proportion of species infected from 22.8% to 32.4%. Thus, whilst *Wolbachia *remained the dominant inherited bacterium, it alone was responsible for around half of all inherited infections of the bacteria sampled, with members of the *Cardinium*, *Arsenophonus *and *Spiroplasma ixodetis *clades each occurring in 4% to 7% of all species. The observation that infection was sometimes rare within host populations, and that there was variation in presence of symbionts between populations indicates that our survey will itself underscore incidence.

**Conclusion:**

This extensive survey demonstrates that at least a third of arthropod species are infected by a diverse assemblage of maternally inherited bacteria that are likely to strongly influence their hosts' biology, and indicates an urgent need to establish the nature of the interaction between non-*Wolbachia *bacteria and their hosts.

## Background

The last 20 years have witnessed an explosion of interest in reproductive parasites: maternally inherited microbial infections of arthropods that manipulate the reproduction of their host species towards the production or survival of infected female hosts. This interest has come largely from the study of one bacterium, *Wolbachia*. This bacterium was found to be associated with a variety of different manipulations: forcing asexuality on its host, feminising hosts, inducing incompatibility between infected males and uninfected (or differently infected) females, killing males and, most recently, being required for oogenesis [1-4]. Each of these manipulations has been fascinating in its own right, and is also of ecological and evolutionary importance to the particular host species that is infected, potentially inducing reproductive isolation, driving changes in sexuality, or alteration in reproductive ecology [5-7]. They also engender strong selection against their action, driving some of the fastest natural selection observed in the field [8]. *Wolbachia *was also found to be common [9,10]. Thus, the effects described above in particular species were likely to be widely important.

Until recently, work in the field of reproductive parasitism has been biased towards *Wolbachia*, and the potential importance of inherited parasites has generally been equated with the importance of *Wolbachia*. Within the last five years, a second inherited bacterium, *Cardinium*, has received increasing attention. Found in fewer species than *Wolbachia *[11,12], its list of manipulation phenotypes is almost as impressive [13-20]. However, *Wolbachia *and *Cardinium *are just two of many bacterial lineages known to act as reproductive parasites. To date, five other bacterial reproductive parasites, unrelated to *Wolbachia *and *Cardinium*, have been recorded in the genera *Arsenophonus*, *Rickettsia*, non-*Cardinium *members of the Flavobacterium clade and *Spiroplasma *(listed in Table 1). Manipulation of host reproduction has been demonstrated in at least one member of each of these clades, although in other cases the nature of the interaction with the host is currently uncertain and reproductive parasitism is unlikely [21-23].

**Table 1:** Bacteria with know manipulation of reproductive biology of arthropods

Organism	Phenotype	Host	Reference
α-PROTEOBACTERIA			
*Rickettsia sp.*	P	*Neochrysocharis formosa *(Hymenoptera: Eulophidae)	[64]
	MK	*Brachys tessellatus *(Coleoptera: Buprestidae)	[65]
		*Adalia bipunctata *(Coleoptera: Coccinellidae)	[66]
		*Adalia decempunctata *(Coleoptera: Coccinellidae)	[67]
*Wolbachia pipientis*	CI	*Culex pipiens *(Diptera: Culicidae)*	[68]
	F	*Armadillidium vulgare *(Isopoda: Armidillidiidae)*	[32]
	P	*Trichogramma *spp (Hymenoptera: Pteromelidae)*	[32,69]
	MK	*Acraea encedon *(Lepidoptera: Nymphalidae)*	[59]
	Oogenesis	*Asobara tabida *(Hymenoptera: Braconidae)*	[1]
γ-PROTEOBACTERIA			
*Arsenophonus nasoniae*	MK	*Nasonia vitripennis *(Hymenoptera: Pteromelidae)	[62]
BACTEROIDETES			
*Cardinium hertigii*	CI	*Encarsia pergendiella *(Hymenoptera: Aphelinidae)	[14]
		*Eotetranychus suginamensis *(Acari: Tetranychidae)	[13]
	F	*Brevipalpus *spp (Acari: Tenuipalpidae)	[17]
	P	*Encarsia *ssp (Hymenoptera: Aphelinidae)	[19,20]
		*Aspidiotus nerii *(Hemiptera: Diaspididae)	[16]
*Flavobacterium sp.*	MK	*Adonia variegata *(Coleoptera: Coccinellidae)	[70]
		*Coleomegilla maculata *(Coleoptera: Coccinellidae)	[38]
MOLLICUTES			
*Spiroplasma ixodetis*	MK	*Adalia bipunctata *(Coleoptera: Coccinellidae)	[56]
		*Anisosticta novemdecimpunctata *(Coleoptera: Coccinellidae)	[57]
		*Harmonia axyridis *(Coleoptera: Coccinellidae)	[71]
		*Danaus chrysippus *(Lepidoptera: Nymphalidae)	[58]
*S. poulsonii*	MK	*Drosophila willistoni *group (Diptera: Drosophilidae)	[41]

CI, cytoplasmic incompatibility; F, feminisation of genetic males; P, thelytokous parthenogenesis; MK, male killing. *The list of *Wolbachia *hosts is not exhaustive and only the species from which an effect was discovered first is reported.

The incidence of *Wolbachia *[9,10], and more recently *Cardinium *[11,12], have received much attention in studies of arthropod biology. However, the incidence of the broader spectrum of inherited bacteria associated with reproductive parasitism is not known, apart from focussed studies of spiders [22,24] and *Drosophila *[25]. In addition, a generally low sampling intensity per species for which screens have taken place in previous surveys may have resulted in the underscoring of *Wolbachia *and *Cardinium *infections, as some reproductive parasite infections are rare within a host species [22,26,27]. The global diversity of reproductive parasites in their natural contexts and the nature of the interactions with their hosts thus remain to be characterised in depth. Here we test a range of arthropods for the presence of the seven known reproductive parasite clades within the eubacteria, estimating incidence, prevalence and geographical variation between natural populations of arthropods. We sample each species more intensively than in past surveys; that is, primarily more than 10 individuals per species. Lastly, we estimate the relatedness of bacterial strains from our collection to strains recorded previously. We conclude that two other inherited bacteria, members of the clade *Arsenophonus *and members of the *Spiroplasma ixodetis *clade, are important associates of insects and demand further study.

## Methods

### Arthropod collection

Specimens belonging to major families of terrestrial arthropods were collected from various field sites, principally in Western Europe (2004–2006). Species were identified based on the morphology of specimens, and the sex was identified wherever possible through genital morphology. The species screened and their origins are presented in Additional file 1. Most of the spider collection came from a previous study [22]. One population per species was generally analysed, except for seven species for which two to three distinct populations were collected. Within each population there were 1 to 25 individuals separately investigated for analysis. Arthropods were fixed in 95% ethanol and stored at 4°C until analysed. Specimens were separated during storage so as to avoid any potential inter-individual transmission.

### Screening and sequencing

Arthropod DNA was extracted using the PROMEGA Wizard^® ^SV 96 Genomic DNA Purification System following the instructions of the manufacturer. DNA extraction was performed on the entire body or abdominal tissue rather than legs, to reduce the risk of missing infection with reproductive parasites when they are present (false negatives). The DNA quality was systematically tested using polymerase chain reaction (PCR) amplification of a conserved region of the arthropod *18S rDNA *using primers listed in Table 2. Infections have been investigated in each individual host for seven reproductive parasites: spotted fever group *Rickettsia*, *Wolbachia*, *Arsenophonus*, *Cardinium*, male killers (MKs) within the flavobacteria, *S. ixodetis *and *S. poulsonii*. Independent assays for infection by each reproductive parasite were performed using PCR amplification of a fragment of either the *16S rDNA *gene or the *17-kDa ompA *gene using specific primers (Table 2). Infected-positive individuals were used as positive controls in each PCR assay (Table 2). Additional PCR amplifications of the *Wolbachia *surface protein gene (*wsp*) were completed in some cases to differentiate the diversity of strains present (primers listed in Table 2). PCRs were performed under the following conditions: initial denaturation at 95°C for 2 minutes, 35 cycles of denaturation (94°C, 30 seconds), annealing (50 to 55°C, depending on primers, 30 seconds), extension (72°C, 1 minute to 1 minute 30 seconds) and a final extension at 72°C for 5 minutes. The PCR products were electrophoresed in a 1.5% agarose gel. Where a PCR product was obtained, this was sequenced from two randomly sampled individuals per infected species to ensure that the record represented a true positive and not a PCR artefact or related bacterium. PCR products were sequenced directly (through both strands) and analysed using the Basic Local Alignment Search Tool (BLAST) to establish that they were within the target clade. The relationship between strains was then estimated. Sequences were first aligned and modified visually using MEGA version 3.1 [28]. Phylogenetic analyses were conducted by the neighbour-joining (NJ) method and the maximum-parsimony (MP) method using MEGA version 3.1 [28]. NJ phylogenies were constructed based upon unambiguously aligned sites using the Tajima-Nei model of nucleotide substitution [29]. MP phylogenies were constructed using the close-neighbour-interchange method [30]. Bootstrap probabilities were calculated by generating 1000 bootstrap replicates. The sequences are deposited in GenBank (accession numbers EU333926–EU333941 and EU727094–EU727140).

**Table 2:** Genes and primers used in polymerase chain reaction (PCR) assays to detect reproductive parasites and control DNA quality

Organism	Gene	Primer (5' – 3')	Annealing temperature	Positive control	Size	Reference
Arthropods	*18S rDNA*	NSF4/18 – CTGGTTGATYCTGCCAGT *	54°C	Any arthropod	400–450 bp	[72,73]
		NSR399/19 – TCTCAGGCTCCYTCTCCGG *				
*Rickettsia sp.*	*17-kDa*	R1 – GCTCTTGCAACTTCTATGTT *	54°C	*Adalia decempunctata*	434 bp	[67]
		R2 – CATTGTTCGTCAGGTTGGCG *				
*Wolbachia pipientis*	*16S rDNA*	16Swolb76-99f – TTGTAGCCTGCTATGGTATAACT *	54°C	*Culex pipiens*	896 bp	[74]
		16Swolb1012-994r – GAATAGGTATGATTTTCATGA *				
	*wsp*	81F – TGGTCCAATAAGTGATGAAGAAAC	50°C	81F/522R: *Aedes albopictus *– *w*AlbB	442 bp	[75]
		136F – TGAAATTTTACCTCTTTTC		172F/691R: *Aedes albopictus *– *w*AlbA	514 bp	
		172F – ACCTATAAGAAAGACAAG		81F/484R: *Protocalliphora sp. *– *w*A2	380 bp	
		484R – TTTGATCATTCACAGCGT		136F/691R: *Protocalliphora sp. *– *w*A1	516 bp	
		522R – ACCAGCTTTTGCTTGATA				
		691R – AAAAATTAAACGCTACTCCA				
*Arsenophonus nasoniae*	*16S rDNA*	ArsF – GGGTTGTAAAGTACTTTCAGTCGT *	52°C	ArsF, ArsF3/ArsR2, ArsR3: *Nasonia vitripennis*	581–804 bp	This study
		ArsF2 – CCCTAAGCTTAACTTAGGA *		ArsF2/ArsR2: *Polistes nympha*	612 bp	
		ArsF3 – GTCGTGAGGAARGTGTTARGGTT *				
		ArsR2 – GTAGCCCTRCTCGTAAGGGCC *				
		ArsR3 – CCTYTATCTCTAAAGGMTTCGCTGGATG *				
*Cardinium hertigii*	*16S rDNA*	CLO-f1 – GGAACCTTACCTGGGCTAGAATGTATT *	54°C	*Holocnemus pluchei*	CLO-f1/CLO-r1: 466 bp	[13]
		CLO-r1 – GCCACTGTCTTCAAGCTCTACCAAC *			ChF/CLO-r1: 953 bp	
		ChF – TACTGTAAGAATAAGCACCGGC				[12]
*Flavobacterium sp.*	*16S rDNA*	FlavF – CGAATAAGTRTCGGCAAACTCCG *	52°C	*Coleomegilla maculata*	530 bp	This study
		FlavR – CTAAACTRTTTCYAGCTTATTCG *				
*Spiroplasma ixodetis*	*16S rDNA*	SpixoF – TTAGGGGCTCAACCCCTAACC *	52°C	*Adalia bipunctata*	810 bp	This study
		SpixoR – TCTGGCATTGCCAACTCTC *				
*S. poulsonii*	*16S rDNA*	SpoulF – GCTTAACTCCAGTTCGCC *	55°C	*Drosophila melanogaster*	421 bp	[42]
		SpoulR – CCTGTCTCAATGTTAACCTC *				

The sizes of PCR products provided are those of positive controls in base pairs (bp). *Primers used in initial screen of the arthropod collection; the other primers were used to obtain additional sequences.

## Results

### Distribution of infection

We assayed for the presence of 7 putative reproductive parasites in 2052 individual arthropods from 136 species encompassing 115 genera, 36 families, 15 orders and three classes. All host DNA samples retained for analysis were positive for PCR amplification using the 18S rDNA arthropod universal primers, indicating satisfactory DNA template quality (less than 1% of DNA templates failed to amplify the *18S rDNA *fragment by PCR). Of the 136 species examined, PCR assay indicated the occurrence of infections by putative bacterial reproductive parasites in 44 species (32.4%). *Wolbachia *was found to infect 31 species (22.8%), *S. ixodetis *infected 9 species (6.6%), *Arsenophonus *infected 6 species (4.4%), *Cardinium *infected 6 species (4.4%), spotted fever group *Rickettsia *infected 1 species (0.7%) and *S. poulsonii *infected 1 species (0.7%); see Table 3 and Additional file 2.

**Table 3:** Summarised results of the screen of arthropod species for the presence of maternally inherited bacteria

Taxon	Number of species	Number of individuals	Number of infected species (number of infected individuals)							
			
			*Rickettsia*	*Wolbachia*	*Cardinium*	*Arsenophonus*	*Flavobacterium*	*Spiroplasma ixodetis*	*S. poulsonii*	multi-infection
ARACHNIDA										
Araneae	26	516	_	10 (78)	6 (60)	1 (32)	_	5 (41)	1 (12)	5 (22)
Ixodidae	4	77	_	_	_	_	_	_	_	_
Opiliones	1	16	_	_	_	_	_	_	_	_
Scorpiones	1	1	_	_	_	_	_	_	_	_
INSECTA										
Blattaria	1	23	_	_	_	1 (23)	_	_	_	_
Coleoptera	26	401	1 (1)	1 (12)	_	_	_	1 (6)	_	_
Dermaptera	1	16	_	_	_	_	_	_	_	_
Diptera	25	310	_	3 (72)	_	2 (17)	_	1 (6)	_	2 (32)
Hemiptera	21	271	_	7 (104)	_	1 (12)	_	2 (11)	_	2 (11)
Hymenoptera	11	104	_	1 (13)	_	1 (20)	_	_	_	1 (20)
Lepidoptera	7	75	_	3 (29)	_	_	_	_	_	_
Mantodea	2	9	_	_	_	_	_	_	_	_
Odonata	1	21	_	_	_	_	_	_	_	_
Orthoptera	5	72	_	2 (12)	_	_	_	_	_	_
MALACOSTRACA										
Isopoda	4	140	_	3 (46)	_	_	_	_	_	_

TOTAL	136	2052	1 (1)	31 (366)	6 (60)	6 (104)	_	9 (64)	1 (12)	10 (85)

For *Wolbachia*, 29 single infections were observed (as ascertained through direct sequences of PCR products that were easily readable without messy peaks) and two multiple infections (with messy peaks). That these were double infections was ascertained through the observation of a clean sequence using primer pairs that distinguish clades within *Wolbachia*, making a total of 33 strains detected. *Arsenophonus *was also observed as a double infection in one species, *Polistes nimpha*, where direct sequence indicated more than one infection; strains were differentiated using the ArsF3/ArsR3 and ArsF2/ArsR2 pairs of 16S rDNA primers. Thus, seven *Arsenophonus *infections were observed in total. *Cardinium*, *Rickettsia*, *S. ixodetis *and *S. poulsonii *where present were observed solely as single infections. Thus, in total we obtained 57 different strains allied to inherited parasites in our sample, 33 *Wolbachia *and 24 'non-*Wolbachia*'. Co-infection by strains of unrelated bacteria (such as *Wolbachia *and *Cardinium*) was observed in 8 of the 44 infected species (Table 3 and Additional file 2). Adding in the three species co-infected with different strains of the same bacterium, 10 species were finally infected by a diverse assemblage of bacterial strains. However, in only nine species did this reflect co-infection at the individual level.

Note that the assays for flavobacteria, *Arsenophonus *and *Spiroplasma poulsonii *also resulted in false positives for these bacteria (a band was present, but the sequence was outside of the target clade). In the case of the flavobacterium, a member of the beneficial clade of inherited bacteria, *Blattabacterium*, was detected in *Loboptera decipiens*, a cockroach, and a known obligate symbiont in *Icerya purchasi*, a scale insect. In the case of *Arsenophonus*, *Sodalis sp. *was detected in one of the 136 species (0.7%), *Providencia sp. *in two (1.5%), *Pectobacterium sp. *in two (1.5%), *Pantoea sp. *in one (0.7%) and *Serratia sp. *in one (0.7%). Of these, *Sodalis *is known to be an inherited bacterium, and the others are likely to represent gut or environmental contaminants. In the case of the *S. poulsonii *assay, two spiroplasmas from outside the *citri *clade were amplified, whose relationship with the host species is uncertain. These infections were all excluded from further analysis.

We asked four questions of the true positive infections. First, do inherited parasites differ in incidence? Second, are there any host taxa where infection is more or less common than expected? Third, what is the prevalence of infection in different species, and is there any evidence of sex bias in infection prevalence that would indicate sex-ratio distortion activity? Fourth, does narrow geographic sampling cause an underestimation of incidence?

With respect to the first question, *Wolbachia *was found to infect significantly more species than the other maternally inherited bacteria (*P *< 0.001, Fisher's exact test). *Wolbachia *was found in most families of three classes of terrestrial arthropods (Arachnida, Insecta and Malacostraca; Table 3 and Additional file 1). The point estimate of 22.8% of species infected with *Wolbachia *in this study is numerically higher than recorded previously, but still lies close to the range of values previously reported in wide surveys of arthropods (incidence from 16.6% [9] to 19.3% [10]; all *P *> 0.18, Fisher's exact test).

The second question we examined was: are there any host taxa where infection with reproductive parasites is more common? Four orders were sampled with more than 20 species: Araneae, Coleoptera, Diptera and Hemiptera. Infection incidence varied between these taxa (Fisher's exact test, *P *= 0.002). As suggested previously, the Araneae were a particular hotspot (with 16 species infected out of 26; 61.5%) having higher incidence than Coleoptera (*P *= 0.001) and Diptera (*P *= 0.004) but not Hemiptera (*P *= 0.15). However, the three insect clades remain homogeneous (*P *= 0.19), although the Hemiptera (8 out of 21 species infected; 38.1%) has a higher incidence than Coleoptera (4 out of 26; 15%) and Diptera (5 out of 25; 20%). The Araneae (spiders) was a particular hotspot for *Cardinium*, with *Wolbachia *and *S. ixodetis *infections also more common in the spider sample than in those of other arthropods. Notably, results from assays of individuals via templates derived from abdomen were reflected precisely by results from the template derived from legs, indicating that the infections that were detected were internalised within the host, and not derived from the contents of the gut. Despite representing less than 20% of the total species sample, all six *Cardinium *infections were found in this group (Fisher's exact test, *P *< 0.001). Five of the nine *S. ixodetis *infected species and 10 of 30 *Wolbachia *infected species were also in the Araneae, both of which are cases of over-representation (Fisher's exact test incidence in Araneae versus others; *P *< 0.025 in each case). Of the four infections represented more than once in the data set, only *Arsenophonus *was not over-represented in spiders (1 of 26 Araneae species versus 5 of 110 non-Araneae; Fisher's exact test, *P *> 0.5).

The third question we asked related to the prevalence of these bacteria and the effect of screening depth on the incidence discovered. Inherited bacteria exist at a variety of prevalence levels in natural populations, from 1% to 100% of individuals. Where *Wolbachia *infection was observed in a host species, prevalence ranged from rare (5%) to very common (100%) at the population level (Table 4 and Additional file 1). *Wolbachia *infection was generally fixed, or close to fixation, except in spiders where prevalence was more variable (5% to 95%). In *Cardinium*, *S. ixodetis *and *S. poulsonii*-infected species, low and medium prevalence (15% to 75%) was observed in all cases, and infection was never observed in all specimens. In the *Rickettsia*-infected species, only 1 individual (8.3%) out of 12 was positive for infection. In the case of *Arsenophonus*, prevalence reached systematically high values (>75%) and infection was frequently fixed, except in the case of *Arsenophonus *infection in the fly *Protocalliphora sp. *(2 infected individuals out of 12; 16.7%). Aside from the *Protocalliphora *datum, *Arsenophonus *prevalence data contrasted with the original record of *Arsenophonus nasoniae*, which infected just 4% of individuals in the wasp *Nasonia vitripennis *[31].

**Table 4:** Frequency of different prevalence of infection amongst infected species (male and female specimens combined) for 57 strains of maternally inherited bacteria

Prevalence in species	All	*Rickettsia*	*Wolbachia*	*Arsenophonus*	*Cardinium*	*Flavobacterium*	*Spiroplasma ixodetis*	*S. poulsonii*
High (*P ≥ *0.80)	29 (0.51)	_	22 (0.67)	6 (0.86)	_	_	_	_
Medium (0.20 ≥ *P *> 0.80)	22 (0.39)	_	7 (0.21)	_	6 (1.00)	_	8 (0.89)	1 (1.00)
Low (*P *< 0.20)	7 (0.12)	1 (1.00)	4 (0.12)	1 (0.14)	_	_	1 (0.11)	_
Total	57	1	33	7	6	_	9	1

We also examined the data for sex bias in infection prevalence, as an indication of potential sex ratio distorting activity. Bacteria other than *Wolbachia *showed no evidence of sex-biased prevalence (all *P *> 0.08, Fisher's exact test; see Additional file 1). *Wolbachia *did show evidence for sex-biased prevalence in some cases. Three species, *Meta mengei *(Tetragnathidae), *Tetragnatha montana *(Tetragnathidae) and *Armadillidium vulgare *(Armadillidiidae) carried *Wolbachia *infection more commonly in females than males (*P *= 0.001, 0.03 and 0.0001, respectively, Fisher's exact test) but only *M. mengei *and *A. vulgare *displayed a significant difference after sequential Bonferroni correction for multiple comparisons (feminising *Wolbachia *was previously evidenced in *A. vulgare*; compare with [32]). Whilst there was no evidence to reject the null hypothesis of equal prevalence in male and females in other cases, we would note that in all spider species (apart from *Pholcus phalangioides*) and in the moth *Chilo sp. *(Crambidae), *Wolbachia *infection was generally rarer in males than females, or was even absent in males, suggesting more intensive sampling would be useful. Furthermore, males do not exist in the parthenogenetic wasp *Diplolepis rosae *(Cynipidae) and all of the females sampled were *Wolbachia*-positive.

The fourth question related to the presence of geographic variation in incidence that may potentially produce underscoring of incidence where one population alone is sampled. We examined specimens from different locations in seven species (see Additional file 1). Four of the species showed no variation in infection presence between locations: the different populations of *Argiope lobata *(Araneidae) and *Cetonia aurata *(Scarabaeidae) were uninfected in all locations and *P. phalangioides *and *A. vulgare *were infected with the same strain (based on *16S rDNA *sequences) wherever sampled. In contrast, in *Linyphia triangularis *(Linyphiidae), *Cylisticus convexus *(Cylisticidae) and *Porcellio dilatatus *(Porcellionidae), the infection presence varied between the populations sampled. In *C. convexus *and *P. dilatatus*, there was one *Wolbachia*-infected and one uninfected population, which in the case of *P. dilatatus *related to sub-specific status (*P. d. petiti *was infected whereas *P. d. dilatatus *was not; see Additional file 1). In the case of *L. triangularis*, the two United Kingdom populations were infected by *Wolbachia*, but the population from Germany was not (*P *= 0.004, Fisher's exact test; see Additional file 1). *Cardinium *infection was observed to be common in both the United Kingdom and German *L. triangularis *populations, and the prevalence did not differ significantly between populations (*P *= 0.27, Fisher's exact test).

### Phylogenetic analysis

The phylogenetic relationships amongst each group of inherited bacterium was estimated using the sequences obtained in this study (including those of non-inherited bacteria), as well as sequences from other hosts available in GenBank. Topologies of the trees obtained under MP were similar to the NJ trees presented in Additional files 3 to 7. Each bacterial group formed a distinct and robust monophyletic clade of arthropod symbionts. Within each group, very closely related symbionts were frequently found in distantly related arthropod hosts.

Arthropods from this study carried *Wolbachia *strains belonging to four supergroups (A, B, F and G) of the eight currently recognised within the *Wolbachia *clade (A to H; see [33]). Although the *Wolbachia *phylogeny was primarily constructed based on the *16S rDNA *sequences from mono-infected species (Additional file 3A), the phylogeny of the *Wolbachia *strains occurring in multi-infection (such as in *Aedes albopictus *and *Protocalliphora sp.*) have been established using the *wsp *sequences (Additional file 3B). Most *Wolbachia *strains (20 out of 33 strains; 60.6%) belonged to the B supergroup. Six *Wolbachia *strains (18.2%) belonged to the A supergroup, five (15.2%) to the G supergroup and one (3.0%) to the F supergroup. However, the *L. triangularis Wolbachia *could not be attached to any supergroup currently described, although it clustered with the *Wolbachia *strain from *Drosophila takahashii *described previously [25] (Additional file 3A).

The *Cardinium *phylogeny was constructed using the six *Cardinium 16S rDNA *sequences from spiders, as well as *Cardinium *sequences from Hymenoptera, the Hemiptera and the Acari available in GenBank, using the closest known relative of *Cardinium*, the *Acanthamoeba *symbiont *Amoebophilus asiaticus*, as an outgroup (Additional file 4). The spider *Cardinium *strains clearly fall within the *Cardinium *species group and especially with *Cardinium *strains from Acari species. However, the power of the phylogeny to resolve relationships between the strains collected to date is poor, owing to a dearth of informative characters in the slow evolving *16S rDNA *sequence.

Nine *Spiroplasma *strains from diverse origins (Araneae, Diptera and Hemiptera) clustered strongly in the *S. ixodetis *clade, but bootstrapping provided little support to branches within this clade (Additional file 5). The *Spiroplasma *strain of *Pardosa lugubris *(Lycosidae) clustered closely with *S. poulsonii *within the *S. citri *clade. The attachment of the two remaining *Spiroplasma *strains to a *Spiroplasma *clade remained ambiguous, although they are close to the *S. citri *clade.

The *Rickettsia *strain from *Mordellistena sp. *clearly falls among the rickettsias and has highest sequence similarity to the maternally inherited endosymbiont of the cat fleas *Ctenocephalides felis *which belong to the *Rickettsia felis *group [34] (Additional file 6). However, the strain of *Mordellistena. sp. *was highly divergent from the MK *Rickettsia *identified in Coccinellidae.

The *Arsenophonus *strains formed a robust clade of arthropod endosymbionts (Additional file 7). The bacterial strains of *Hippobosca equina *(Hippoboscidae), *Protocalliphora sp. *(Calliphoridae),* Pyrrhocoris apterus *(Pyrrhocoridae) and *Polistes nimpha *(Vespidae) were tightly clustered with the MK *Arsenophonus *strain. The three other *Arsenophonus *strains from *Araneus diadematus *(Araneae), *Loboptera decipiens *(Blatellidae) and *P. nimpha *fitted better with bacterial strains isolated in bloodsucking flies [35] and lice [36]. Although some phylogenetic structure did exist within the *Arsenophonus *clade, it is not well resolved by analysis of the *16S rRNA *gene, although the subgroup which included the MK *Arsenophonus *appears distinct from *Arsenophonus *isolates from bloodsucking flies and lice previously reported [35,36].

## Discussion

We sampled a medium number of male and female individuals (median 20 total) of 136 arthropods species for *Wolbachia*, a reproductive parasite well known to be common in various arthropod taxa [9,10], for *Cardinium*, a reproductive parasite known to exist widely but in fewer species [11,12,22], and for five other unrelated reproductive parasites whose incidence and prevalence were largely unknown. The observed incidence of infection for arthropods was 32.4% for inherited bacteria putatively acting as reproductive parasites.

Our results reveal that *Wolbachia *is, as expected, the most common reproductive parasite clade associated with arthropods, being recorded in 22.8% of species in our sample. Three other clades, *Cardinium*, *S. ixodetis *and *Arsenophonus *bacteria were present widely, with each occurring in 4% to 7% of the arthropod species. Whilst there has been some work on the former of these bacteria, *S. ixodetis *and *Arsenophonus *clearly represent 'understudied groups' that merit careful investigation. We would also note that the frequency of these four bacteria means that around one fifth of infections co-occurred with other species of inherited bacteria (8 of 44 infected species carry two different bacteria), an estimate of course made conservative by our own restrictive sampling for 'known' symbiont groups. This observation reinforces the call of Weeks et al. [37] to adequately sample the inherited flora of a species before interpreting data in terms of particular inherited bacteria.

There were three clades of bacteria that were either found in just one species or not found: *Rickettsia*, *S. poulsonii *and flavobacteria relatives. With respect to flavobacteria, the PCR assay detected allied clades of beneficial bacteria and was thus broad spectrum. We can thus be clear that this bacterial clade does not commonly reach mid or high prevalence outside ladybird beetles, where infection was initially established [38]. It is possible that this bacterial group is phylogenetically restricted to being 'ladybird MK'. The low incidence of *Rickettsia *was more surprising. These have been conjectured as being common, understudied bacterial symbionts of arthropods [39], but we can be confident that spotted fever group *Rickettsia *does not commonly exist at medium to high prevalence. This comment is made with the caveat that our screen for *Rickettsia *was relatively narrow, designed to find bacteria allied to a clade of ladybird MK, and would exclude some *Rickettsia*. Finally, there is *S. poulsonii*, otherwise known as group I spiroplasmas [40]. Other spiroplasmas were detected in the assay, indicating the PCR assay had a relatively broad catch. *S. poulsonii *itself is a MK associate of *Drosophila *species [41], with related strains in leafhoppers. Clearly, whilst spiroplasmas may be common, this particular clade either has low incidence, or always has low prevalence (as is the case for *S. poulsonii *in *Drosophila*, for example [42]).

As there is an intuitive link between the frequency of symbiotic infection and the overall importance of symbionts in arthropod biology, some comment on the true incidence level of these bacteria is appropriate. Given that we have tested against the presence of false positives in the PCR assay by obtaining product sequence in all cases, we would emphasise that our estimate underscores true incidence. Two main sources of underestimation of incidence are inherent in our survey. First, there is the possibility of false negatives in the PCR assay. These would be individuals (and, hence, species) in our sample that are in fact infected, but where infection has not been detected. Second, there are species where the PCR assay was accurate and our samples were truly uninfected, but other members of the species, not sampled and tested, are in fact infected. We would argue that both of these factors lead to a considerable underestimation of the frequency of infection.

False negatives with respect to undetected presence in infected samples are an issue with PCR assays, their sensitivity to titre and their ability to detect diverse infections. For *Wolbachia*, for instance, our use of a PCR assay based on *16S rDNA *is likely to include most of the *Wolbachia *diversity. For *Rickettsia*, we would note that whereas we found no *Rickettsia *infection among spiders, Goodacre et al. [24] used PCR primers with a broader 'catch' and reported 19.7% of species to be infected by *Rickettsia *strains related only distantly to *Rickettsia *MK strains. These are likely to be inherited bacteria, and possibly reproductive parasites, but they would not be detected in our screen. Furthermore, we probably also underestimated the number of bacterial strains present in species we found to be infected. Given that our survey is in most cases based on PCR amplification of fragments of the slow evolving *16S rDNA *sequence, we had limited power to detect multiple infections of closely related bacterial strains. For instance, the *16S rDNA *sequences of *Wolbachia *infecting the mosquito *Culex pipiens *are well known to be strictly identical, suggesting that only one *Wolbachia *strain could occur, but sequencing of faster evolving genes has demonstrated the occurrence of more than 60 *Wolbachia *strains in this host species [43-45].

The second issue that causes an underestimation of the frequency of these agents is failing to sample an infected individual in an infected species. There are two aspects to this, the first being cases where infections are rare within a population. For example, MK infections tend to exist at low prevalence and examples of infection at between 1% and 10% frequency in females represent about one-half of all known infections [46]. Within our survey, we sampled two species in which just 1 of 20 individuals was infected, which clearly indicates the potential for false negatives arising from low prevalence infection. The second cause of failure to sample an infected individual arises from infection presence varying from population to population. We sampled seven species from several localities and revealed three examples of the presence or absence variation between populations within Western Europe. Such geographical variation is not uncommon [47], and is likely to be much greater when the scale of sampling is increased beyond Western Europe (which because of the recent ice age represents one recently recolonised region in ecological terms). Insufficient geographical sampling will lead to serious underestimation of infection incidence.

Overall, therefore, our point estimate of 32.4% of species infected with bacteria allied to reproductive parasites is likely to seriously underestimate the true figure. Our data improve on past surveys by increasing the intensity of sampling within species. For instance, for the case of *Wolbachia*, resampling our data (taking one individual from each species that is infected with a probability of infection given by the overall prevalence found) indicates that the move from single individual sampling to our modest multiple individual sampling reveals around one-third more cases of infection: the median *Wolbachia *incidence on sampling one individual per species is 17.6%, rather than the observed 22.8%. However, the sample size within each species is still limited and, most importantly, the restriction in geographical sampling may produce a very serious underestimation of symbiont presence or absence.

We obtained some insight into the degree to which host taxa vary in the frequency of their interaction with inherited bacteria. The depth of conclusion is very limited by virtue of the intensity of sampling within particular clades in a broad survey. Nevertheless, spiders (the Araneae) harboured a higher richness of inherited bacteria than others and represented diversity hotspots for these bacteria with 61.5% of species infected, contrasting to the relatively low incidence observed within Diptera and Coleoptera. The Araneae hotspot was present for *Wolbachia*, *Cardinium *and *S. ixodetis*, although not for *Arsenophonus*. Why some arthropod taxa are hotspots for inherited bacteria remains one of the most challenging questions with regard to the ecology of reproductive parasites. Certain taxa are clearly more susceptible to acquiring bacteria via horizontal transfer and/or to stably maintaining infection. Given that cocladogenesis is rare, it is the establishment of new infections that appears important in dictating incidence [48]. Intimate contact with other species is likely to predispose to transfer, be this parasitism, predation, physical damage or becoming prey [49-53]. Indeed, species which include other living arthropods in their diet through predation or parasitism are more frequently infected (43.8%) than other species (26.1%; *P *= 0.05, Fisher's exact test). As a result, predation and/or parasitism could be an efficient transfer mechanism to elucidate the origin of hotspots, as illustrated by Araneae, which is exceptional because all species of this group depend completely on predation of other invertebrates which are largely polyphagous [54].

An alternative hypothesis to account for heterogeneity of endosymbiont incidence among host clades is based on differences in host phylogenies [55], but this does not seem to be a likely explanation for the Araneae.

## Conclusion

We hope that this paper has established that *Wolbachia *and *Cardinium *are not alone: *S. ixodetis *and *Arsenophonus *relatives also exist in appreciable numbers of arthropod species. Perhaps the most important consideration for the future is not the incidence of infection, but phenotype. Whilst there have been detailed studies of *Wolbachia *and *Cardinium *in a variety of arthropods, there has been little work on the other pair of 'common' symbionts, *S. ixodetis *relatives and *Arsenophonus*. *S. ixodetis *relatives are known to be MKs in ladybirds [56,57] and butterflies [58,59], but have also been isolated from ticks [60] and aphids [61], where they do not kill males. *A. nasoniae *was established as the MK of the wasp *N. vitripennis *[62] but other strains of *Arsenophonus *isolated from a divergent range of arthropods show no evidence of sex-ratio distortion activity [21,23]. The *S. ixodetis *and *Arsenophonus *strains isolated in our study do not show evidence of MK behaviour as they are present in both males and females. It is very likely that these symbionts represent important understudied components of arthropod biology. We would advocate an open approach to their biology; they may cause cytoplasmic incompatibility, but may also be secondary symbionts that could be conditionally beneficial, and their presence in bacterial clades containing reproductive parasites would be of great interest. It is also possible that horizontal (infectious) transmission plays a larger role in the population biology of these infections than is usually perceived. Sexual transmission of aphid secondary symbionts [63] and transmission on coparasitisation for *Arsenophonus *[31] make this avenue worthy of investigation. If biologists are to understand the role that inherited bacteria play in arthropod evolution, a thorough examination with respect to documenting their effects is now required.

## Authors' contributions

OD carried out the molecular genetic studies, the sequence alignment and drafted the manuscript. DB, SB, LB, LZ and JE participated in the molecular genetic studies. OD and GDH conceived of the study, and participated in its design and coordination. All authors read and approved the final manuscript.

## Supplementary Material

Additional file 1 Table showing detailed results of the screen of arthropods for inherited bacteria. Prevalence of infection is given overall, for females and for males. Difference in prevalence between sexes was tested using Fisher's exact test (*P < 0.05; **P < 0.01; ***P < 0.001). Only *M. mengei *and *A. vulgare *displayed a significant difference in prevalence after a Bonferroni correction for multiple comparisons. na, not ascertained; ov., overall; un., undetermined. Click here for file

Additional file 2 Table showing results of arthropod screening for species with co-infection, detailing frequency of co-infection. Only *M. mengei *displayed a significant difference between sexes in *Wolbachia *prevalence after a Bonferroni correction. na, not ascertained; ov., overall; un., undetermined. Click here for file

Additional file 3 *Wolbachia *16S rDNA (A) and *wsp *(B) phylogenies constructed via neighbour-joining as implemented on MEGA version 3.1. The symbionts have the prefix S followed by the proper name of their host. Sequences from this study are underlined and some previously published *Wolbachia *sequences are shown in plain type. Effect of infection is indicated in bold type if known (CI, cytoplasmic incompatibility; P, parthenogenesis; F, feminisation). Major *Wolbachia *supergroup lineages are reported (A)-(H). Numbers on branches indicate percentage bootstrap support for major branches (1000 replicates; only bootstrap values of 60% or more are shown). Click here for file

Additional file 4 *Cardinium *16S rDNA phylogeny constructed via neighbour-joining as implemented on MEGA version 3.1. The symbionts have the prefix S followed by the proper name of their host, whereas free-living bacteria have proper binomial nomenclature. Sequences from this study are underlined and some previously published *Cardinium *sequences are shown in plain type. Effect of infection is indicated in bold type if known (CI, cytoplasmic incompatibility; P, parthenogenesis). The closest known relative of *Cardinium*, the *Acanthamoeba *symbiont *Amoebophilus asiaticus*, was used as an outgroup. Numbers on branches indicate percentage bootstrap support for major branches (1000 replicates; only bootstrap values of 60% or more are shown). Click here for file

Additional file 5 *Spiroplasma *16S rDNA phylogeny constructed via neighbour-joining as implemented on MEGA version 3.1. The symbionts have the prefix S followed by the proper name of their host. Sequences from this study are underlined and some previously published *Spiroplasma *sequences are shown in plain type. Effect of infection on host reproduction is indicated in bold type if known (MK, male killing). The closest known relative of *Spiroplasma*, the human pathogen *Mycoplasma hominis*, was used as an outgroup. Numbers on branches indicate percentage bootstrap support for major branches (1000 replicates; only bootstrap values of 60% or more are shown). Click here for file

Additional file 6 *Rickettsia *17 kDa *ompA *gene phylogeny constructed via neighbour-joining as implemented on MEGA version 3.1. The symbionts have the prefix S followed by the proper name of their host. Sequences from this study are underlined and some previously published *Rickettsia *sequences are shown in plain type. Effect of infection on host reproduction is indicated in bold type if known (MK, male killing). Numbers on branches indicate percentage bootstrap support for major branches (1000 replicates; only bootstrap values of 60% or more are shown). Click here for file

Additional file 7 *Arsenophonus *16S rDNA phylogeny constructed via neighbour-joining as implemented on MEGA version 3.1. The symbionts have the prefix S followed by the proper name of their host. Symbionts of *Pediculus *species have been previously given a different name (*Riesia spp.*, cf. [36]). Sequences from this study are underlined and some previously published *Arsenophonus *sequences are shown in plain type. Effect of infection on host reproduction is indicated in bold type if known (MK, male killing). Some related bacteria to the *Arsenophonus *clade have been also included. Numbers on branches indicate percentage bootstrap support for major branches (1000 replicates; only bootstrap values of 60% or more are shown). Click here for file
