# Supplementary material for: The diversity of reproductive parasites among arthropods: Wolbachia do not walk alone
Source: BMC Biol. 2008 Jun 24;6:27. doi: 10.1186/1741-7007-6-27 (PMC2492848; doi:10.1186/1741-7007-6-27)
Supplement: Additional file 1 — Table showing detailed results of the screen of arthropods for inherited bacteria. Prevalence of infection is given overall, for females and for males. Difference in prevalence between sexes was tested using Fisher's exact test (*P < 0.05; **P < 0.01; ***P < 0.001). Only M. mengei and A. vulgare displayed a significant difference in prevalence after a Bonferroni correction for multiple comparisons. na, not ascertained; ov., overall; un., undetermined. [file 1741-7007-6-27-S1.doc]

| Taxon | | Population | Sample size | | | *Rickettsia* | | | *Wolbachia* | | | *Arsenophonus* | | | *Cardinium* | | | *Flavobacterium* | | | *Spiroplasma ixodetis* | | | *S. poulsonii* | | |
| --- | --- | --- | --- | --- | --- | --- | --- | --- | --- | --- | --- | --- | --- | --- | --- | --- | --- | --- | --- | --- | --- | --- | --- | --- | --- | --- |
|  |  |  | ♂ | ♀ | un. | ov. | ♂ | ♀ | ov. | ♂ | ♀ | ov. | ♂ | ♀ | ov. | ♂ | ♀ | ov. | ♂ | ♀ | ov. | ♂ | ♀ | ov. | ♂ | ♀ |
|  |  |  |  |  |  |  |  |  |  |  |  |  |  |  |  |  |  |  |  |  |  |  |  |  |  |  |
| **ARACHNIDA** | |  |  |  |  |  |  |  |  |  |  |  |  |  |  |  |  |  |  |  |  |  |  |  |  |  |
|  | **ARANEAE** |  |  |  |  |  |  |  |  |  |  |  |  |  |  |  |  |  |  |  |  |  |  |  |  |  |
|  | ARANEOMORPHAE |  |  |  |  |  |  |  |  |  |  |  |  |  |  |  |  |  |  |  |  |  |  |  |  |  |
|  | **Amaurobiidae** |  |  |  |  |  |  |  |  |  |  |  |  |  |  |  |  |  |  |  |  |  |  |  |  |  |
|  | *Amaurobius fenestralis* | Montpellier, France, 2006 | 6 | 10 | - | - | - | - | - | - | - | - | - | - | - | - | - | - | - | - | - | - | - | - | - | - |
|  | **Araneidae** |  |  |  |  |  |  |  |  |  |  |  |  |  |  |  |  |  |  |  |  |  |  |  |  |  |
|  | *Agelenopsis aperta* | Tennessee, USA, 2005 | 10 | 10 | - | - | - | - | - | - | - | - | - | - | - | - | - | - | - | - | - | - | - | - | - | - |
|  | *Araneus diadematus* | Beerse, Belgium, 2005 | 10 | 10 | - | - | - | - | - | - | - | 0.85 | 0.70 | 1.00 | - | - | - | - | - | - | 0.35 | 0.50 | 0.20 | - | - | - |
|  |  | London, UK, 2005 | 3 | 5 | - | - | - | - | - | - | - | 0.75 | 0.67 | 0.80 | - | - | - | - | - | - | 0.25 | 0.00 | 0.40 | - | - | - |
|  | *Argiope bruennichi* | Hamburg, Germany, 2005 | 10 | 10 | - | - | - | - | - | - | - | - | - | - | - | - | - | - | - | - | - | - | - | - | - | - |
|  | *Argiope lobota* | Israel, 2005 | 5 | 4 | - | - | - | - | - | - | - | - | - | - | - | - | - | - | - | - | - | - | - | - | - | - |
|  |  | Spain, 2005 | 3 | 4 | - | - | - | - | - | - | - | - | - | - | - | - | - | - | - | - | - | - | - | - | - | - |
|  | *Cyclosa conica* | Berlin, Germany, 2004-05 | 10 | 10 | - | - | - | - | - | - | - | - | - | - | 0.40 | 0.60 | 0.20 | - | - | - | - | - | - | - | - | - |
|  | *Larinioides cornutus* | London, UK, 2005 | 10 | 10 | - | - | - | - | - | - | - | - | - | - | - | - | - | - | - | - | - | - | - | - | - | - |
|  | *Larinioides sclopetarius* | Hamburg, Germany, 2005 | 10 | 10 | - | - | - | - | - | - | - | - | - | - | - | - | - | - | - | - | - | - | - | - | - | - |
|  | **Dysderidae** |  |  |  |  |  |  |  |  |  |  |  |  |  |  |  |  |  |  |  |  |  |  |  |  |  |
|  | *Dysdera crocata* | Montpellier, France, 2006 | 1 | 1 | - | - | - | - | - | - | - | - | - | - | - | - | - | - | - | - | - | - | - | - | - | - |
|  | **Linyphiidae** |  |  |  |  |  |  |  |  |  |  |  |  |  |  |  |  |  |  |  |  |  |  |  |  |  |
|  | *Linyphia triangularis* | Berlin, Germany, 2004 | 1 | 8 | - | - | - | - | - | - | - | - | - | - | 0.50 | 1.00 | 0.38 | - | - | - | - | - | - | - | - | - |
|  |  | London (Richmond Park), UK, 2004-05 | 1 | 7 | - | - | - | - | 0.38 | 0.00 | 0.43 | - | - | - | 0.63 | 1.00 | 0.57 | - | - | - | - | - | - | - | - | - |
|  |  | London (Trent Park), UK, 2004-05 | 2 | 7 | - | - | - | - | 0.78 | 0.00 | 0.88 | - | - | - | 0.25 | 0.50 | 0.14 | - | - | - | - | - | - | - | - | - |
|  | *Nereine clathrata* | Beerse, Belgium, 2005 | 10 | 10 | - | - | - | - | - | - | - | - | - | - | - | - | - | - | - | - | 0.15 | 0.10 | 0.20 | - | - | - |
|  | **Lycosidae** |  |  |  |  |  |  |  |  |  |  |  |  |  |  |  |  |  |  |  |  |  |  |  |  |  |
|  | *Alopecosa pulverulenta* | Bern, Switzerland, 2005 | 10 | 10 | - | - | - | - | 0.25 | 0.10 | 0.40 | - | - | - | 0.75 | 0.70 | 0.80 | - | - | - | - | - | - | - | - | - |
|  | *Pardosa lugubris* | Darmstadt, Germany, 2005 | 10 | 10 | - | - | - | - | - | - | - | - | - | - | - | - | - | - | - | - | - | - | - | 0.60 | 0.60 | 0.60 |
|  | *Pardosa pullata* | Bern, Switzerland, 2005 | 10 | 10 | - | - | - | - | 0.05 | 0.10 | 0.00 | - | - | - | - | - | - | - | - | - | - | - | - | - | - | - |
|  | *Pardosa purbeckensis* | Salt march, Belgium, 2005 | 10 | 10 | - | - | - | - | - | - | - | - | - | - | - | - | - | - | - | - | - | - | - | - | - | - |
|  | **Pholcidae** |  |  |  |  |  |  |  |  |  |  |  |  |  |  |  |  |  |  |  |  |  |  |  |  |  |
|  | *Holocnemus pluchei* | Montpellier, France, 2006 | 10 | 10 | - | - | - | - | - | - | - | - | - | - | 0.40 | 0.60 | 0.20 | - | - | - | - | - | - | - | - | - |
|  | *Pholcus phalangioides* | Berlin, Germany, 20-05 | 10 | 10 | - | - | - | - | 0.90 | 0.90 | 0.90 | - | - | - | - | - | - | - | - | - | - | - | - | - | - | - |
|  |  | London, 2007 | 10 | 10 | - | - | - | - | 0.95 | 0.90 | 1.00 | - | - | - | - | - | - | - | - | - | - | - | - | - | - | - |
|  | **Pisauridae** |  |  |  |  |  |  |  |  |  |  |  |  |  |  |  |  |  |  |  |  |  |  |  |  |  |
|  | *Pisaura mirabilis* | London, UK, 2004 | 2 | 10 | - | - | - | - | - | - | - | - | - | - | - | - | - | - | - | - | - | - | - | - | - | - |
|  | **Salticidae** |  |  |  |  |  |  |  |  |  |  |  |  |  |  |  |  |  |  |  |  |  |  |  |  |  |
|  | *Evarcha falcata* | Beerse, Belgium, 2005 | 10 | 10 | - | - | - | - | - | - | - | - | - | - | 0.45 | 0.40 | 0.50 | - | - | - | - | - | - | - | - | - |
|  | **Tetragnathidae** |  |  |  |  |  |  |  |  |  |  |  |  |  |  |  |  |  |  |  |  |  |  |  |  |  |
|  | *Meta mengei* | London, UK, 2005 | 10 | 10 | - | - | - | - | 0.50 | 0.10 | 0.90** | - | - | - | - | - | - | - | - | - | 0.65 | 0.80 | 0.50 | - | - | - |
|  | *Meta segmenta* | Berlin, Germany, 2004 | 10 | 10 | - | - | - | - | 0.15 | 0.10 | 0.20 | - | - | - | - | - | - | - | - | - | 0.60 | 0.80 | 0.40 | - | - | - |
|  | *Pachygnatha degeerri* | Bern, Switzerland, 2004 | 10 | 10 | - | - | - | - | 0.05 | 0.00 | 0.10 | - | - | - | 0.45 | 0.40 | 0.50 | - | - | - | - | - | - | - | - | - |
|  | *Pachygnatha listeri* | Beerse, Belgium, 2005 | 10 | 10 | - | - | - | - | 0.25 | 0.20 | 0.30 | - | - | - | - | - | - | - | - | - | - | - | - | - | - | - |
|  | *Tetragnatha montana* | London, UK, 2005 | 10 | 10 | - | - | - | - | 0.25 | 0.00 | 0.50* | - | - | - | - | - | - | - | - | - | 0.20 | 0.30 | 0.10 | - | - | - |
|  | **Theridiidae** |  |  |  |  |  |  |  |  |  |  |  |  |  |  |  |  |  |  |  |  |  |  |  |  |  |
|  | *Enoplognatha ovata* | London, UK, 2004-2005 | 10 | 10 | - | - | - | - | 0.05 | 0.00 | 0.10 | - | - | - | - | - | - | - | - | - | - | - | - | - | - | - |
|  | *Xysticus cristatus* | Cambridge, UK, 2005 | 8 | 8 | - | - | - | - | - | - | - | - | - | - | - | - | - | - | - | - | - | - | - | - | - | - |
|  |  |  |  |  |  |  |  |  |  |  |  |  |  |  |  |  |  |  |  |  |  |  |  |  |  |  |
|  | **IXODIDA** |  |  |  |  |  |  |  |  |  |  |  |  |  |  |  |  |  |  |  |  |  |  |  |  |  |
|  | **Ixodidae** |  |  |  |  |  |  |  |  |  |  |  |  |  |  |  |  |  |  |  |  |  |  |  |  |  |
|  | *Ixodes ricinus* | Switzerland, 1996 | - | - | 20 | - | - | - | - | - | - | - | - | - | - | - | - | - | - | - | - | - | - | - | - | - |
|  | *Ixodes uriae* | Hornøya, Norway, 2005 | 10 | 10 | - | - | - | - | - | - | - | - | - | - | - | - | - | - | - | - | - | - | - | - | - | - |
|  | *Rhipicephalus evertsi* | Zimbabwe | - | 12 | - | - | - | - | - | - | - | - | - | - | - | - | - | - | - | - | - | - | - | - | - | - |
|  | *Rhipicephalus microplus* | New Caledonia, 2003 | 5 | 20 | - | - | - | - | - | - | - | - | - | - | - | - | - | - | - | - | - | - | - | - | - | - |
|  |  |  |  |  |  |  |  |  |  |  |  |  |  |  |  |  |  |  |  |  |  |  |  |  |  |  |
|  | **OPIOLONES** |  |  |  |  |  |  |  |  |  |  |  |  |  |  |  |  |  |  |  |  |  |  |  |  |  |
|  | PALPATORES |  |  |  |  |  |  |  |  |  |  |  |  |  |  |  |  |  |  |  |  |  |  |  |  |  |
|  | **Leiobunidae** |  |  |  |  |  |  |  |  |  |  |  |  |  |  |  |  |  |  |  |  |  |  |  |  |  |
|  | *Leiobunum rotundum* | Feurs, France, 2006 | 10 | 6 | - | - | - | - | - | - | - | - | - | - | - | - | - | - | - | - | - | - | - | - | - | - |
|  |  |  |  |  |  |  |  |  |  |  |  |  |  |  |  |  |  |  |  |  |  |  |  |  |  |  |
|  | **SCORPIONES** |  |  |  |  |  |  |  |  |  |  |  |  |  |  |  |  |  |  |  |  |  |  |  |  |  |
|  | **Chactidae** |  |  |  |  |  |  |  |  |  |  |  |  |  |  |  |  |  |  |  |  |  |  |  |  |  |
|  | *Euscorpius flavicauda* | St Nazaire de Pézan, France, 2006 | - | - | 1 | - | - | - | - | - | - | - | - | - | - | - | - | - | - | - | - | - | - | - | - | - |
|  |  |  |  |  |  |  |  |  |  |  |  |  |  |  |  |  |  |  |  |  |  |  |  |  |  |  |
| **INSECTA** | |  |  |  |  |  |  |  |  |  |  |  |  |  |  |  |  |  |  |  |  |  |  |  |  |  |
|  | **BLATTARIA** |  |  |  |  |  |  |  |  |  |  |  |  |  |  |  |  |  |  |  |  |  |  |  |  |  |
|  | **Blatellidae** |  |  |  |  |  |  |  |  |  |  |  |  |  |  |  |  |  |  |  |  |  |  |  |  |  |
|  | *Loboptera decipiens* | Montpellier, France, 2006 | - | - | 23 | - | - | - | - | - | - | 1.00 | na | na | - | - | - | - | - | - | - | - | - | - | - | - |
|  |  |  |  |  |  |  |  |  |  |  |  |  |  |  |  |  |  |  |  |  |  |  |  |  |  |  |
|  | **COLEOPTERA** |  |  |  |  |  |  |  |  |  |  |  |  |  |  |  |  |  |  |  |  |  |  |  |  |  |
|  | ADEPHAGA |  |  |  |  |  |  |  |  |  |  |  |  |  |  |  |  |  |  |  |  |  |  |  |  |  |
|  | **Dytiscidae** |  |  |  |  |  |  |  |  |  |  |  |  |  |  |  |  |  |  |  |  |  |  |  |  |  |
|  | *Guignotus pusillus* | Notre Dame de Londres, France, 2006 | - | - | 12 | - | - | - | 1.00 | na | na | - | - | - | - | - | - | - | - | - | - | - | - | - | - | - |
|  | POLYPHAGA |  |  |  |  |  |  |  |  |  |  |  |  |  |  |  |  |  |  |  |  |  |  |  |  |  |
|  | **Buprestidae** |  |  |  |  |  |  |  |  |  |  |  |  |  |  |  |  |  |  |  |  |  |  |  |  |  |
|  | *Anthaxia nitidula* | Mont Barri, France, 2004 | 10 | 9 | - | - | - | - | - | - | - | - | - | - | - | - | - | - | - | - | - | - | - | - | - | - |
|  | *Anthaxia sp.* | Mont Barri, France, 2004 | 10 | 6 | - | - | - | - | - | - | - | - | - | - | - | - | - | - | - | - | - | - | - | - | - | - |
|  | *Capnodis tenebrionis* | Montpellier, France, 2006 | - | - | 3 | - | - | - | - | - | - | - | - | - | - | - | - | - | - | - | - | - | - | - | - | - |
|  | **Cantharidae** |  |  |  |  |  |  |  |  |  |  |  |  |  |  |  |  |  |  |  |  |  |  |  |  |  |
|  | *Rhagonycha fulva* | London, UK, 2006 | 7 | 7 | - | - | - | - | - | - | - | - | - | - | - | - | - | - | - | - | - | - | - | - | - | - |
|  | *Rhagonycha sp.* | London, UK, 2006 | 12 | 12 | - | - | - | - | - | - | - | - | - | - | - | - | - | - | - | - | - | - | - | - | - | - |
|  | **Cerambycidae** |  |  |  |  |  |  |  |  |  |  |  |  |  |  |  |  |  |  |  |  |  |  |  |  |  |
|  | *Clytus arietis* | Mont Barri, France, 2004 | 10 | 9 | - | - | - | - | - | - | - | - | - | - | - | - | - | - | - | - | - | - | - | - | - | - |
|  | *Leptura livida* | Mont Barri, France, 2004 | 10 | 10 | - | - | - | - | - | - | - | - | - | - | - | - | - | - | - | - | - | - | - | - | - | - |
|  | *Stenopterus sp.* | Mont Barri, France, 2004 | 10 | 10 | - | - | - | - | - | - | - | - | - | - | - | - | - | - | - | - | - | - | - | - | - | - |
|  | **Chrysomelidae** |  |  |  |  |  |  |  |  |  |  |  |  |  |  |  |  |  |  |  |  |  |  |  |  |  |
|  | *Chrysolina varians* | Mont Barri, France, 2004 | 10 | 9 | - | - | - | - | - | - | - | - | - | - | - | - | - | - | - | - | 0.32 | 0.30 | 0.33 | - | - | - |
|  | *Leptinotarsa decemlineata* | Feurs, France, 2006 | - | - | 12 | - | - | - | - | - | - | - | - | - | - | - | - | - | - | - | - | - | - | - | - | - |
|  | unidentified species | London, UK, 2006 | 12 | 8 | - | - | - | - | - | - | - | - | - | - | - | - | - | - | - | - | - | - | - | - | - | - |
|  | **Coccinellidae** |  |  |  |  |  |  |  |  |  |  |  |  |  |  |  |  |  |  |  |  |  |  |  |  |  |
|  | *Calvia 14-guttata* | Hampstead (London), UK, 2006 | - | - | 9 | - | - | - | - | - | - | - | - | - | - | - | - | - | - | - | - | - | - | - | - | - |
|  | *Propylea 14-punctata* | Hammersmith (London), UK, 2006 | 12 | 11 | - | - | - | - | - | - | - | - | - | - | - | - | - | - | - | - | - | - | - | - | - | - |
|  | *Psyllobora 22-punctata* | Hammersmith (London), UK, 2006 | - | - | 12 | - | - | - | - | - | - | - | - | - | - | - | - | - | - | - | - | - | - | - | - | - |
|  | *Tytthaspis 16-punctata* | Richmond Park (London), UK, 2006 | 7 | 12 | - | - | - | - | - | - | - | - | - | - | - | - | - | - | - | - | - | - | - | - | - | - |
|  | **Curculionidae** |  |  |  |  |  |  |  |  |  |  |  |  |  |  |  |  |  |  |  |  |  |  |  |  |  |
|  | *Larinus cynarae* | Sardaigne, 1999 | 8 | 9 | - | - | - | - | - | - | - | - | - | - | - | - | - | - | - | - | - | - | - | - | - | - |
|  | *Larinus scolymi* | Aldira de Irmeros, Spain, 2005 | - | - | 12 | - | - | - | - | - | - | - | - | - | - | - | - | - | - | - | - | - | - | - | - | - |
|  | *Phyllobius argentatus* | Mont Barri, France, 2004 | 7 | 10 | - | - | - | - | - | - | - | - | - | - | - | - | - | - | - | - | - | - | - | - | - | - |
|  | **Dermestidae** |  |  |  |  |  |  |  |  |  |  |  |  |  |  |  |  |  |  |  |  |  |  |  |  |  |
|  | *Dermestes sp.* | Mont Barri, France, 2004 | 10 | 10 | - | - | - | - | - | - | - | - | - | - | - | - | - | - | - | - | - | - | - | - | - | - |
|  | **Lucanidae** |  |  |  |  |  |  |  |  |  |  |  |  |  |  |  |  |  |  |  |  |  |  |  |  |  |
|  | *Geotrupes stercorarius* | Mont Barri, France, 2004 | - | - | 3 | - | - | - | - | - | - | - | - | - | - | - | - | - | - | - | - | - | - | - | - | - |
|  | **Mordellidae** |  |  |  |  |  |  |  |  |  |  |  |  |  |  |  |  |  |  |  |  |  |  |  |  |  |
|  | *Mordellistena sp.* | Mont Barri, France, 2004 | - | - | 12 | 0.08 | na | na | - | - | - | - | - | - | - | - | - | - | - | - | - | - | - | - | - | - |
|  | **Oedemeridae** |  |  |  |  |  |  |  |  |  |  |  |  |  |  |  |  |  |  |  |  |  |  |  |  |  |
|  | *Oncocerna sp.* | Mont Barri, France, 2004 | 10 | 10 | - | - | - | - | - | - | - | - | - | - | - | - | - | - | - | - | - | - | - | - | - | - |
|  | **Scarabaeidae** |  |  |  |  |  |  |  |  |  |  |  |  |  |  |  |  |  |  |  |  |  |  |  |  |  |
|  | *Cetonia aurata* | Feurs, France, 2006 | - | - | 3 | - | - | - | - | - | - | - | - | - | - | - | - | - | - | - | - | - | - | - | - | - |
|  |  | Mont Barri, France, 2004 | - | - | 12 | - | - | - | - | - | - | - | - | - | - | - | - | - | - | - | - | - | - | - | - | - |
|  | **Scraptiidae** |  |  |  |  |  |  |  |  |  |  |  |  |  |  |  |  |  |  |  |  |  |  |  |  |  |
|  | *Anaspis frontalis* | Mont Barri, France, 2004 | - | - | 12 | - | - | - | - | - | - | - | - | - | - | - | - | - | - | - | - | - | - | - | - | - |
|  | **Tenebrionidae** |  |  |  |  |  |  |  |  |  |  |  |  |  |  |  |  |  |  |  |  |  |  |  |  |  |
|  | *Tenebrio molitor* | UK, 2006 | - | - | 12 | - | - | - | - | - | - | - | - | - | - | - | - | - | - | - | - | - | - | - | - | - |
|  |  |  |  |  |  |  |  |  |  |  |  |  |  |  |  |  |  |  |  |  |  |  |  |  |  |  |
|  | **DERMAPTERA** |  |  |  |  |  |  |  |  |  |  |  |  |  |  |  |  |  |  |  |  |  |  |  |  |  |
|  | FORFICULINA |  |  |  |  |  |  |  |  |  |  |  |  |  |  |  |  |  |  |  |  |  |  |  |  |  |
|  | **Forficulidae** |  |  |  |  |  |  |  |  |  |  |  |  |  |  |  |  |  |  |  |  |  |  |  |  |  |
|  | *Forficula auricularia* | Feurs, France, 2006 | 6 | 10 | - | - | - | - | - | - | - | - | - | - | - | - | - | - | - | - | - | - | - | - | - | - |
|  |  |  |  |  |  |  |  |  |  |  |  |  |  |  |  |  |  |  |  |  |  |  |  |  |  |  |
|  | **DIPTERA** |  |  |  |  |  |  |  |  |  |  |  |  |  |  |  |  |  |  |  |  |  |  |  |  |  |
|  | BRACHYCERA |  |  |  |  |  |  |  |  |  |  |  |  |  |  |  |  |  |  |  |  |  |  |  |  |  |
|  | **Anthomyiidae** |  |  |  |  |  |  |  |  |  |  |  |  |  |  |  |  |  |  |  |  |  |  |  |  |  |
|  | *Delia antiqua* | France | 4 | 7 | - | - | - | - | - | - | - | - | - | - | - | - | - | - | - | - | - | - | - | - | - | - |
|  | *Delia platura* | France | - | - | 11 | - | - | - | - | - | - | - | - | - | - | - | - | - | - | - | - | - | - | - | - | - |
|  | *Delia radicum* | France | 4 | 6 | - | - | - | - | - | - | - | - | - | - | - | - | - | - | - | - | - | - | - | - | - | - |
|  | **Braulidae** |  |  |  |  |  |  |  |  |  |  |  |  |  |  |  |  |  |  |  |  |  |  |  |  |  |
|  | *Braula coeca* | Ouessant, France, 2002 | - | - | 4 | - | - | - | - | - | - | - | - | - | - | - | - | - | - | - | - | - | - | - | - | - |
|  | **Calliphoridae** |  |  |  |  |  |  |  |  |  |  |  |  |  |  |  |  |  |  |  |  |  |  |  |  |  |
|  | *Protocalliphora  sp.* | Corse, 2003 | - | - | 12 | - | - | - | 1.00 | na | na | 0.17 | na | na | - | - | - | - | - | - | - | - | - | - | - | - |
|  | **Coelopidae** |  |  |  |  |  |  |  |  |  |  |  |  |  |  |  |  |  |  |  |  |  |  |  |  |  |
|  | *Coelopa pilipes* | France | - | - | 7 | - | - | - | - | - | - | - | - | - | - | - | - | - | - | - | - | - | - | - | - | - |
|  | **Dolichopodidae** |  |  |  |  |  |  |  |  |  |  |  |  |  |  |  |  |  |  |  |  |  |  |  |  |  |
|  | *Medetera petrophila* | St Bauzille de Putois, France, 2003 | - | - | 12 | - | - | - | - | - | - | - | - | - | - | - | - | - | - | - | - | - | - | - | - | - |
|  | **Gasterophilidae** |  |  |  |  |  |  |  |  |  |  |  |  |  |  |  |  |  |  |  |  |  |  |  |  |  |
|  | *Gasterophilus intestinalis* | France | - | - | 12 | - | - | - | - | - | - | - | - | - | - | - | - | - | - | - | - | - | - | - | - | - |
|  | **Hippoboscidae** |  |  |  |  |  |  |  |  |  |  |  |  |  |  |  |  |  |  |  |  |  |  |  |  |  |
|  | *Hippobosca equina* | Restinclières, France, 2006 | 7 | 8 | - | - | - | - | - | - | - | 1.00 | na | na | - | - | - | - | - | - | - | - | - | - | - | - |
|  | **Lonchopteridae** |  |  |  |  |  |  |  |  |  |  |  |  |  |  |  |  |  |  |  |  |  |  |  |  |  |
|  | *Lonchoptera lutea* | La Faute sur Mer, France, 2003 | - | - | 10 | - | - | - | - | - | - | - | - | - | - | - | - | - | - | - | - | - | - | - | - | - |
|  | **Muscidae** |  |  |  |  |  |  |  |  |  |  |  |  |  |  |  |  |  |  |  |  |  |  |  |  |  |
|  | *Musca vitripennis* | Notre Dame de Londres, France, 2003 | - | - | 8 | - | - | - | - | - | - | - | - | - | - | - | - | - | - | - | - | - | - | - | - | - |
|  | *Musca domestica* | L'Olme, France, 2006 | 8 | 12 | - | - | - | - | - | - | - | - | - | - | - | - | - | - | - | - | - | - | - | - | - | - |
|  | *Neomyia cornicina* | Notre Dame de Londres, France, 2003 | - | - | 8 | - | - | - | - | - | - | - | - | - | - | - | - | - | - | - | - | - | - | - | - | - |
|  | *Stomoxys calcitrans* | Le Malzieu, France, 2001 | - | - | 11 | - | - | - | - | - | - | - | - | - | - | - | - | - | - | - | - | - | - | - | - | - |
|  | **Psilidae** |  |  |  |  |  |  |  |  |  |  |  |  |  |  |  |  |  |  |  |  |  |  |  |  |  |
|  | *Psila rosae* | France | - | - | 11 | - | - | - | - | - | - | - | - | - | - | - | - | - | - | - | - | - | - | - | - | - |
|  | **Simuliidae** |  |  |  |  |  |  |  |  |  |  |  |  |  |  |  |  |  |  |  |  |  |  |  |  |  |
|  | *Simulium ornatum* | France, 2003 | - | - | 12 | - | - | - | - | - | - | - | - | - | - | - | - | - | - | - | - | - | - | - | - | - |
|  | **Stratiomyidae** |  |  |  |  |  |  |  |  |  |  |  |  |  |  |  |  |  |  |  |  |  |  |  |  |  |
|  | *Chorisops tunisiae* | Montpellier, France, 2004 | - | - | 8 | - | - | - | - | - | - | - | - | - | - | - | - | - | - | - | - | - | - | - | - | - |
|  | **Syrphidae** |  |  |  |  |  |  |  |  |  |  |  |  |  |  |  |  |  |  |  |  |  |  |  |  |  |
|  | *Eristalis tenax* | Montpellier, France, 2002 | - | - | 7 | - | - | - | - | - | - | - | - | - | - | - | - | - | - | - | - | - | - | - | - | - |
|  | **Tephritidae** |  |  |  |  |  |  |  |  |  |  |  |  |  |  |  |  |  |  |  |  |  |  |  |  |  |
|  | *Ceratitis capitata* | Montpellier, France, 2003 | 3 | 8 | - | - | - | - | - | - | - | - | - | - | - | - | - | - | - | - | - | - | - | - | - | - |
|  | unidentified species | London, UK, 2006 | 9 | 8 | - | - | - | - | - | - | - | - | - | - | - | - | - | - | - | - | - | - | - | - | - | - |
|  | NEMATOCERA |  |  |  |  |  |  |  |  |  |  |  |  |  |  |  |  |  |  |  |  |  |  |  |  |  |
|  | **Chironomidae** |  |  |  |  |  |  |  |  |  |  |  |  |  |  |  |  |  |  |  |  |  |  |  |  |  |
|  | *Chironomus sp.* | Notre Dame de Londres, France, 2006 | 10 | 10 | - | - | - | - | - | - | - | - | - | - | - | - | - | - | - | - | - | - | - | - | - | - |
|  | **Culicidae** |  |  |  |  |  |  |  |  |  |  |  |  |  |  |  |  |  |  |  |  |  |  |  |  |  |
|  | *Aedes albopictus* | Roma, Italia, 2005 | 10 | 10 | - | - | - | - | 1.00 | 1.00 | 1.00 | - | - | - | - | - | - | - | - | - | - | - | - | - | - | - |
|  | *Culex pipiens* | St Nazaire de Pézan, France, 2006 | 10 | 10 | - | - | - | - | 1.00 | 1.00 | 1.00 | - | - | - | - | - | - | - | - | - | - | - | - | - | - | - |
|  | *Culex quinquefasciatus* | Puerto Viejo de Talamanca, Costa Rica, 2006 | 10 | 10 | - | - | - | - | 1.00 | 1.00 | 1.00 | - | - | - | - | - | - | - | - | - | - | - | - | - | - | - |
|  | **Tipulidae** |  |  |  |  |  |  |  |  |  |  |  |  |  |  |  |  |  |  |  |  |  |  |  |  |  |
|  | *Tipula oleracea* | UK, 2006 | 8 | 5 | - | - | - | - | - | - | - | - | - | - | - | - | - | - | - | - | 0.58 | 0.40 | 1.00 | - | - | - |
|  |  |  |  |  |  |  |  |  |  |  |  |  |  |  |  |  |  |  |  |  |  |  |  |  |  |  |
|  | **HEMIPTERA** |  |  |  |  |  |  |  |  |  |  |  |  |  |  |  |  |  |  |  |  |  |  |  |  |  |
|  | EUHEMIPTERA |  |  |  |  |  |  |  |  |  |  |  |  |  |  |  |  |  |  |  |  |  |  |  |  |  |
|  | **Acanthosomatidae** |  |  |  |  |  |  |  |  |  |  |  |  |  |  |  |  |  |  |  |  |  |  |  |  |  |
|  | *Elasmucha grisea* | Hammersmith (London), UK, 2006 | 4 | 12 | - | - | - | - | - | - | - | - | - | - | - | - | - | - | - | - | - | - | - | - | - | - |
|  | **Cicadellidae** |  |  |  |  |  |  |  |  |  |  |  |  |  |  |  |  |  |  |  |  |  |  |  |  |  |
|  | *Cicadella viridis* | L'Olme, France, 2006 | 7 | 10 | - | - | - | - | 0.94 | 0.86 | 1.00 | - | - | - | - | - | - | - | - | - | 0.41 | 0.29 | 0.50 | - | - | - |
|  | **Cimicidae** |  |  |  |  |  |  |  |  |  |  |  |  |  |  |  |  |  |  |  |  |  |  |  |  |  |
|  | *Cimex lectularius* | UK, 2006 | - | - | 12 | - | - | - | 1.00 | na | na | - | - | - | - | - | - | - | - | - | - | - | - | - | - | - |
|  | **Corixidae** |  |  |  |  |  |  |  |  |  |  |  |  |  |  |  |  |  |  |  |  |  |  |  |  |  |
|  | *Sigara lateralis* | Notre Dame de Londres, France, 2006 | - | - | 12 | - | - | - | 1.00 | na | na | - | - | - | - | - | - | - | - | - | - | - | - | - | - | - |
|  | **Gerridae** |  |  |  |  |  |  |  |  |  |  |  |  |  |  |  |  |  |  |  |  |  |  |  |  |  |
|  | *Gerris sp.* | Montferrier sur Lez, France, 2006 | - | - | 12 | - | - | - | - | - | - | - | - | - | - | - | - | - | - | - | - | - | - | - | - | - |
|  | **Hydrometridae** |  |  |  |  |  |  |  |  |  |  |  |  |  |  |  |  |  |  |  |  |  |  |  |  |  |
|  | *Hydrometra stagnorum* | Montferrier sur Lez, France, 2006 | 10 | 10 | - | - | - | - | 1.00 | 1.00 | 1.00 | - | - | - | - | - | - | - | - | - | - | - | - | - | - | - |
|  | **Lygaeidae** |  |  |  |  |  |  |  |  |  |  |  |  |  |  |  |  |  |  |  |  |  |  |  |  |  |
|  | *Lygaeus equestris* | Montpellier, France, 2006 | - | - | 12 | - | - | - | - | - | - | - | - | - | - | - | - | - | - | - | - | - | - | - | - | - |
|  | *Rhyparochromus vulgaris* | Castelnaudary, France, 2006 | 10 | 10 | - | - | - | - | 1.00 | 1.00 | 1.00 | - | - | - | - | - | - | - | - | - | - | - | - | - | - | - |
|  | **Miridae** |  |  |  |  |  |  |  |  |  |  |  |  |  |  |  |  |  |  |  |  |  |  |  |  |  |
|  | *Notostira elongata* | L'Olme, France, 2006 | - | - | 12 | - | - | - | 1.00 | na | na | - | - | - | - | - | - | - | - | - | 0.35 | na | na | - | - | - |
|  | **Nepidae** |  |  |  |  |  |  |  |  |  |  |  |  |  |  |  |  |  |  |  |  |  |  |  |  |  |
|  | *Nepa cinerea* | Montferrier sur Lez, France, 2006 | - | - | 3 | - | - | - | - | - | - | - | - | - | - | - | - | - | - | - | - | - | - | - | - | - |
|  | **Pentatomidae** |  |  |  |  |  |  |  |  |  |  |  |  |  |  |  |  |  |  |  |  |  |  |  |  |  |
|  | *Graphosoma italicum* | Montpellier, France, 2006 | - | - | 12 | - | - | - | - | - | - | - | - | - | - | - | - | - | - | - | - | - | - | - | - | - |
|  | **Pleidae** |  |  |  |  |  |  |  |  |  |  |  |  |  |  |  |  |  |  |  |  |  |  |  |  |  |
|  | *Plea minutissima* | Notre Dame de Londres, France, 2006 | - | - | 8 | - | - | - | - | - | - | - | - | - | - | - | - | - | - | - | - | - | - | - | - | - |
|  | **Pyrrhocoridae** |  |  |  |  |  |  |  |  |  |  |  |  |  |  |  |  |  |  |  |  |  |  |  |  |  |
|  | *Pyrrhocoris apterus* | Montpellier, France, 2006 | - | - | 12 | - | - | - | - | - | - | 1.00 | na | na | - | - | - | - | - | - | - | - | - | - | - | - |
|  | STERNORRHYNCHA |  |  |  |  |  |  |  |  |  |  |  |  |  |  |  |  |  |  |  |  |  |  |  |  |  |
|  | **Aphididae** |  |  |  |  |  |  |  |  |  |  |  |  |  |  |  |  |  |  |  |  |  |  |  |  |  |
|  | *Aphis fabae* | Montpellier, France, 2006 | - | 12 | - | - | - | - | - | - | - | - | - | - | - | - | - | - | - | - | - | - | - | - | - | - |
|  | *Aphis nerii* | Montpellier, France, 2006 | - | 16 | - | - | - | - | - | - | - | - | - | - | - | - | - | - | - | - | - | - | - | - | - | - |
|  | **Coccidae** |  |  |  |  |  |  |  |  |  |  |  |  |  |  |  |  |  |  |  |  |  |  |  |  |  |
|  | *Coccus sp.* | Montpellier, France, 2006 | - | 12 | - | - | - | - | - | - | - | - | - | - | - | - | - | - | - | - | - | - | - | - | - | - |
|  | *Saissetia oleae* | Montpellier, France, 2006 | - | 16 | - | - | - | - | - | - | - | - | - | - | - | - | - | - | - | - | - | - | - | - | - | - |
|  | **Diaspididae** |  |  |  |  |  |  |  |  |  |  |  |  |  |  |  |  |  |  |  |  |  |  |  |  |  |
|  | *Lepidosaphes ulmi* | St Nazaire de Pézan, France, 2006 | - | 11 | - | - | - | - | - | - | - | - | - | - | - | - | - | - | - | - | - | - | - | - | - | - |
|  | **Margarodidae** |  |  |  |  |  |  |  |  |  |  |  |  |  |  |  |  |  |  |  |  |  |  |  |  |  |
|  | *Icerya purchasi* | St Nazaire de Pézan, France, 2006 | - | 12 | - | - | - | - | 1.00 | na | 1.00 | - | - | - | - | - | - | - | - | - | - | - | - | - | - | - |
|  | **Pemphigidae** |  |  |  |  |  |  |  |  |  |  |  |  |  |  |  |  |  |  |  |  |  |  |  |  |  |
|  | *Baizongia pistaciae* | Viols le Fort, France, 2006 | - | 12 | - | - | - | - | - | - | - | - | - | - | - | - | - | - | - | - | - | - | - | - | - | - |
|  | **Pseudococcidae** |  |  |  |  |  |  |  |  |  |  |  |  |  |  |  |  |  |  |  |  |  |  |  |  |  |
|  | *Pseudococcus longispinus* | Montpellier, France, 2006 | - | 12 | - | - | - | - | - | - | - | - | - | - | - | - | - | - | - | - | - | - | - | - | - | - |
|  |  |  |  |  |  |  |  |  |  |  |  |  |  |  |  |  |  |  |  |  |  |  |  |  |  |  |
|  | **HYMENOPTERA** |  |  |  |  |  |  |  |  |  |  |  |  |  |  |  |  |  |  |  |  |  |  |  |  |  |
|  | ACULEATA |  |  |  |  |  |  |  |  |  |  |  |  |  |  |  |  |  |  |  |  |  |  |  |  |  |
|  | **Apidae** |  |  |  |  |  |  |  |  |  |  |  |  |  |  |  |  |  |  |  |  |  |  |  |  |  |
|  | *Amegilla albigena* | St Nazaire de Pézan, France, 2006 | 3 | 10 | - | - | - | - | - | - | - | - | - | - | - | - | - | - | - | - | - | - | - | - | - | - |
|  | *Amegilla ochroleuca* | St Nazaire de Pézan, France, 2006 | - | 3 | - | - | - | - | - | - | - | - | - | - | - | - | - | - | - | - | - | - | - | - | - | - |
|  | *Apis mellifera* | UK, 2006 | - | 9 | - | - | - | - | - | - | - | - | - | - | - | - | - | - | - | - | - | - | - | - | - | - |
|  | *Bombus terrestris* | Switzerland, 2006 | 10 | 10 | - | - | - | - | - | - | - | - | - | - | - | - | - | - | - | - | - | - | - | - | - | - |
|  | **Formicidae** |  |  |  |  |  |  |  |  |  |  |  |  |  |  |  |  |  |  |  |  |  |  |  |  |  |
|  | *Formica lugubris* | UK, 2006 | - | 12 | - | - | - | - | - | - | - | - | - | - | - | - | - | - | - | - | - | - | - | - | - | - |
|  | **Megachilidae** |  |  |  |  |  |  |  |  |  |  |  |  |  |  |  |  |  |  |  |  |  |  |  |  |  |
|  | *Anthidium florentinum* | St Nazaire de Pézan, France, 2006 | 2 | 4 | - | - | - | - | - | - | - | - | - | - | - | - | - | - | - | - | - | - | - | - | - | - |
|  | **Sphecidea** |  |  |  |  |  |  |  |  |  |  |  |  |  |  |  |  |  |  |  |  |  |  |  |  |  |
|  | *Sceliphron caementarium* | St Nazaire de Pézan, France, 2006 | - | 3 | - | - | - | - | - | - | - | - | - | - | - | - | - | - | - | - | - | - | - | - | - | - |
|  | **Vespidae** |  |  |  |  |  |  |  |  |  |  |  |  |  |  |  |  |  |  |  |  |  |  |  |  |  |
|  | *Polistes dominulus* | St Nazaire de Pézan, France, 2006 | - | 4 | - | - | - | - | - | - | - | - | - | - | - | - | - | - | - | - | - | - | - | - | - | - |
|  | *Polistes nimpha* | St Nazaire de Pézan, France, 2006 | 10 | 10 | - | - | - | - | - | - | - | 1.00 | 1.00 | 1.00 | - | - | - | - | - | - | - | - | - | - | - | - |
|  | APOCRITA |  |  |  |  |  |  |  |  |  |  |  |  |  |  |  |  |  |  |  |  |  |  |  |  |  |
|  | **Cynipidae** |  |  |  |  |  |  |  |  |  |  |  |  |  |  |  |  |  |  |  |  |  |  |  |  |  |
|  | *Diplolepis rosae* | L'Olme, France, 2006 | - | 13 | - | - | - | - | 1.00 | na | 1.00 | - | - | - | - | - | - | - | - | - | - | - | - | - | - | - |
|  | **Ichneumonidae** |  |  |  |  |  |  |  |  |  |  |  |  |  |  |  |  |  |  |  |  |  |  |  |  |  |
|  | *Amblyteles armatorius* | St Nazaire de Pézan, France, 2006 | - | - | 1 | - | - | - | - | - | - | - | - | - | - | - | - | - | - | - | - | - | - | - | - | - |
|  |  |  |  |  |  |  |  |  |  |  |  |  |  |  |  |  |  |  |  |  |  |  |  |  |  |  |
|  | **LEPIDOPTERA** |  |  |  |  |  |  |  |  |  |  |  |  |  |  |  |  |  |  |  |  |  |  |  |  |  |
|  | GLOSSATA |  |  |  |  |  |  |  |  |  |  |  |  |  |  |  |  |  |  |  |  |  |  |  |  |  |
|  | **Actiidae** |  |  |  |  |  |  |  |  |  |  |  |  |  |  |  |  |  |  |  |  |  |  |  |  |  |
|  | *Euplagia quadripunctaria* | Feurs, France, 2006 | - | - | 2 | - | - | - | - | - | - | - | - | - | - | - | - | - | - | - | - | - | - | - | - | - |
|  | **Crambidae** |  |  |  |  |  |  |  |  |  |  |  |  |  |  |  |  |  |  |  |  |  |  |  |  |  |
|  | *Chilo  sp.* | Feurs, France, 2006 | 3 | 9 | - | - | - | - | 0.25 | 0.00 | 0.33 | - | - | - | - | - | - | - | - | - | - | - | - | - | - | - |
|  | **Hepialidae** |  |  |  |  |  |  |  |  |  |  |  |  |  |  |  |  |  |  |  |  |  |  |  |  |  |
|  | *Triodia sylvina* | Montpellier, France, 2006 | 2 | 2 | - | - | - | - | 1.00 | 1.00 | 1.00 | - | - | - | - | - | - | - | - | - | - | - | - | - | - | - |
|  | **Hesperiidae** |  |  |  |  |  |  |  |  |  |  |  |  |  |  |  |  |  |  |  |  |  |  |  |  |  |
|  | *Thymelicus lineola* | Cockfosters, UK, 2006 | 11 | 4 | - | - | - | - | - | - | - | - | - | - | - | - | - | - | - | - | - | - | - | - | - | - |
|  | *Thymelicus sylvestris* | Cockfosters, UK, 2006 | 10 | 13 | - | - | - | - | 0.96 | 0.90 | 1.00 | - | - | - | - | - | - | - | - | - | - | - | - | - | - | - |
|  | **Pieridae** |  |  |  |  |  |  |  |  |  |  |  |  |  |  |  |  |  |  |  |  |  |  |  |  |  |
|  | *Pieris brassicae* | Feurs, France, 2006 | - | - | 7 | - | - | - | - | - | - | - | - | - | - | - | - | - | - | - | - | - | - | - | - | - |
|  | **Pyralidae** |  |  |  |  |  |  |  |  |  |  |  |  |  |  |  |  |  |  |  |  |  |  |  |  |  |
|  | *Plodia interpunctella* | Montpellier, France, 2006 | - | - | 12 | - | - | - | - | - | - | - | - | - | - | - | - | - | - | - | - | - | - | - | - | - |
|  |  |  |  |  |  |  |  |  |  |  |  |  |  |  |  |  |  |  |  |  |  |  |  |  |  |  |
|  | **MANTODAE** |  |  |  |  |  |  |  |  |  |  |  |  |  |  |  |  |  |  |  |  |  |  |  |  |  |
|  | **Mantidae** |  |  |  |  |  |  |  |  |  |  |  |  |  |  |  |  |  |  |  |  |  |  |  |  |  |
|  | *Iris oratoria* | St Nazaire de Pézan, France, 2006 | 1 | - | 5 | - | - | - | - | - | - | - | - | - | - | - | - | - | - | - | - | - | - | - | - | - |
|  | *Mantis religiosa* | Feurs, France, 2006 | 1 | 2 | - | - | - | - | - | - | - | - | - | - | - | - | - | - | - | - | - | - | - | - | - | - |
|  |  |  |  |  |  |  |  |  |  |  |  |  |  |  |  |  |  |  |  |  |  |  |  |  |  |  |
|  | **ODONATA** |  |  |  |  |  |  |  |  |  |  |  |  |  |  |  |  |  |  |  |  |  |  |  |  |  |
|  | **Coenagrionidae** |  |  |  |  |  |  |  |  |  |  |  |  |  |  |  |  |  |  |  |  |  |  |  |  |  |
|  | unidentified species | Hampstead park, London (UK), 2006 | 11 | 10 | - | - | - | - | - | - | - | - | - | - | - | - | - | - | - | - | - | - | - | - | - | - |
|  |  |  |  |  |  |  |  |  |  |  |  |  |  |  |  |  |  |  |  |  |  |  |  |  |  |  |
|  | **ORTHOPTERA** |  |  |  |  |  |  |  |  |  |  |  |  |  |  |  |  |  |  |  |  |  |  |  |  |  |
|  | CAELIFERA |  |  |  |  |  |  |  |  |  |  |  |  |  |  |  |  |  |  |  |  |  |  |  |  |  |
|  | **Acrididae** |  |  |  |  |  |  |  |  |  |  |  |  |  |  |  |  |  |  |  |  |  |  |  |  |  |
|  | *Calliptamus italicus* | Notre Dame de Londres, France, 2006 | 10 | 8 | - | - | - | - | - | - | - | - | - | - | - | - | - | - | - | - | - | - | - | - | - | - |
|  | *Chorthippus brunneus* | UK, 2006 | 6 | 14 | - | - | - | - | - | - | - | - | - | - | - | - | - | - | - | - | - | - | - | - | - | - |
|  | *Stenobothrus lineatus* | Richmond Park (London), UK, 2006 | 6 | 4 | - | - | - | - | 1.00 | 1.00 | 1.00 | - | - | - | - | - | - | - | - | - | - | - | - | - | - | - |
|  | **Tettigoniidae** |  |  |  |  |  |  |  |  |  |  |  |  |  |  |  |  |  |  |  |  |  |  |  |  |  |
|  | *Metrioptera roeselii* | Richmond Park (London), UK, 2006 | 14 | 8 | - | - | - | - | - | - | - | - | - | - | - | - | - | - | - | - | - | - | - | - | - | - |
|  | ENSIFERA |  |  |  |  |  |  |  |  |  |  |  |  |  |  |  |  |  |  |  |  |  |  |  |  |  |
|  | **Gryllidae** |  |  |  |  |  |  |  |  |  |  |  |  |  |  |  |  |  |  |  |  |  |  |  |  |  |
|  | *Gryllomorpha dalmatina* | Montpellier, France, 2006 | 1 | 1 | - | - | - | - | 1.00 | 1.00 | 1.00 | - | - | - | - | - | - | - | - | - | - | - | - | - | - | - |
|  |  |  |  |  |  |  |  |  |  |  |  |  |  |  |  |  |  |  |  |  |  |  |  |  |  |  |
| **MALACOSTRACA** | |  |  |  |  |  |  |  |  |  |  |  |  |  |  |  |  |  |  |  |  |  |  |  |  |  |
|  | **ISOPODA** |  |  |  |  |  |  |  |  |  |  |  |  |  |  |  |  |  |  |  |  |  |  |  |  |  |
|  | **Armidillidiidae** |  |  |  |  |  |  |  |  |  |  |  |  |  |  |  |  |  |  |  |  |  |  |  |  |  |
|  | *Armadillidium vulgare* | Saint Cyr, France | 10 | 10 | - | - | - | - | 0.20 | 0.00 | 0.40 | - | - | - | - | - | - | - | - | - | - | - | - | - | - | - |
|  |  | Mery sur Cher, France | 10 | 10 | - | - | - | - | 0.45 | 0.00 | 0.90*** | - | - | - | - | - | - | - | - | - | - | - | - | - | - | - |
|  | **Balloniscidae** |  |  |  |  |  |  |  |  |  |  |  |  |  |  |  |  |  |  |  |  |  |  |  |  |  |
|  | *Balloniscus sellowii* | Caxias do Sol, Brazil | 10 | 10 | - | - | - | - | - | - | - | - | - | - | - | - | - | - | - | - | - | - | - | - | - | - |
|  | **Cylisticidae** |  |  |  |  |  |  |  |  |  |  |  |  |  |  |  |  |  |  |  |  |  |  |  |  |  |
|  | *Cylisticus convexus* | Villedaigne, France | 10 | 10 | - | - | - | - | - | - | - | - | - | - | - | - | - | - | - | - | - | - | - | - | - | - |
|  |  | Avanton, France | 10 | 10 | - | - | - | - | 0.80 | 0.70 | 0.90 | - | - | - | - | - | - | - | - | - | - | - | - | - | - | - |
|  | **Porcellionidae** |  |  |  |  |  |  |  |  |  |  |  |  |  |  |  |  |  |  |  |  |  |  |  |  |  |
|  | *Porcellio dilatatus dilatatus* | Rom, France | 11 | 9 | - | - | - | - | - | - | - | - | - | - | - | - | - | - | - | - | - | - | - | - | - | - |
|  | *Porcellio dilatatus petiti* | Saint Honora, France | 10 | 10 | - | - | - | - | 0.80 | 0.70 | 0.90 | - | - | - | - | - | - | - | - | - | - | - | - | - | - | - |
|  |  |  |  |  |  |  |  |  |  |  |  |  |  |  |  |  |  |  |  |  |  |  |  |  |  |  |

**Table S1.** Detailed results of the screen of arthropods for inherited bacteria. Prevalence of infection is given overall, for females, and for males. Difference in prevalence between sexes was tested using Fisher’s exact test (*, *P* < 0.05; **, *P* < 0.01; ***, *P* < 0.001). Only *M. mengei* and *A. vulgare* displayed a significant difference in prevalence after a Bonferroni correction for multiple comparisons. na, not ascertained; ov., overall; un., undetermined.
