# Supplementary material for: The diversity of reproductive parasites among arthropods: Wolbachia do not walk alone
Source: BMC Biol. 2008 Jun 24;6:27. doi: 10.1186/1741-7007-6-27 (PMC2492848; doi:10.1186/1741-7007-6-27)
Supplement: Additional file 2 — Table showing results of arthropod screening for species with co-infection, detailing frequency of co-infection. Only M. mengei displayed a significant difference between sexes in Wolbachia prevalence after a Bonferroni correction. na, not ascertained; ov., overall; un., undetermined. [file 1741-7007-6-27-S2.doc]

| Taxon | | Population | Sample size | | | Infection (cf. Table S1) | | | | Coinfection | | | |
| --- | --- | --- | --- | --- | --- | --- | --- | --- | --- | --- | --- | --- | --- |
|  |  |  | ♂ | ♀ | un. | Bacterial strain | ov. | ♂ | ♀ | Bacterial strains | ov. | ♂ | ♀ |
|  |  |  |  |  |  |  |  |  |  |  |  |  |  |
| **ARACHNIDA** | |  |  |  |  |  |  |  |  |  |  |  |  |
|  | **ARANEAE** |  |  |  |  |  |  |  |  |  |  |  |  |
|  | ARANEOMORPHAE |  |  |  |  |  |  |  |  |  |  |  |  |
|  | **Araneidae** |  |  |  |  |  |  |  |  |  |  |  |  |
|  | *Araneus diadematus* | Beerse, Belgium, 2005 | 10 | 10 | - | *Arsenophonus* | 0.85 | 0.70 | 1.00 | *Arsenophonus / S. ixodetis* | 0.30 | 0.40 | 0.20 |
|  |  |  |  |  |  | *S. ixodetis* | 0.35 | 0.50 | 0.20 |  |  |  |  |
|  |  | London, UK, 2005 | 3 | 5 | - | *Arsenophonus* | 0.75 | 0.67 | 0.80 | *Arsenophonus / S. ixodetis* | 0.12 | 0.00 | 0.20 |
|  |  |  |  |  |  | *S. ixodetis* | 0.25 | 0.00 | 0.40 |  |  |  |  |
|  | **Linyphiidae** |  |  |  |  |  |  |  |  |  |  |  |  |
|  | *Linyphia triangularis* | London (Richmond Park), UK, 2004-05 | 1 | 7 | - | *Wolbachia* | 0.38 | 0.00 | 0.43 | *Wolbachia / Cardinium* | 0.00 | 0.00 | 0.00 |
|  |  |  |  |  |  | *Cardinium* | 0.63 | 1.00 | 0.57 |  |  |  |  |
|  |  | London (Trent Park), UK, 2004-05 | 2 | 7 | - | *Wolbachia* | 0.78 | 0.00 | 0.88 | *Wolbachia / Cardinium* | 0.00 | 0.00 | 0.00 |
|  |  |  |  |  |  | *Cardinium* | 0.25 | 0.50 | 0.14 |  |  |  |  |
|  | **Lycosidae** |  |  |  |  |  |  |  |  |  |  |  |  |
|  | *Alopecosa pulverulenta* | Bern, Switzerland, 2005 | 10 | 10 | - | *Wolbachia* | 0.25 | 0.10 | 0.40 | *Wolbachia / Cardinium* | 0.25 | 0.10 | 0.40 |
|  |  |  |  |  |  | *Cardinium* | 0.75 | 0.70 | 0.80 |  |  |  |  |
|  | **Tetragnathidae** |  |  |  |  |  |  |  |  |  |  |  |  |
|  | *Meta mengei* | London, UK, 2005 | 10 | 10 | - | *Wolbachia* | 0.50 | 0.10 | 0.90** | *Wolbachia / S. ixodetis* | 0.25 | 0.00 | 0.50* |
|  |  |  |  |  |  | *S. ixodetis* | 0.65 | 0.80 | 0.50 |  |  |  |  |
|  | *Meta segmenta* | Berlin, Germany, 2004 | 10 | 10 | - | *Wolbachia* | 0.15 | 0.10 | 0.20 | *Wolbachia / S. ixodetis* | 0.15 | 0.10 | 0.20 |
|  |  |  |  |  |  | *S. ixodetis* | 0.60 | 0.80 | 0.40 |  |  |  |  |
|  |  |  |  |  |  |  |  |  |  |  |  |  |  |
| **INSECTA** | |  |  |  |  |  |  |  |  |  |  |  |  |
|  | **DIPTERA** |  |  |  |  |  |  |  |  |  |  |  |  |
|  | BRACHYCERA |  |  |  |  |  |  |  |  |  |  |  |  |
|  | **Calliphoridae** |  |  |  |  |  |  |  |  |  |  |  |  |
|  | *Protocalliphora  sp.* | Corse, 2003 | - | - | 12 | *Wolbachia* (*w*A1 strain) | 1.00 | na | na | *Wolbachia w*A1 */ Wolbachia w*A2 | 1.00 | na | na |
|  |  |  |  |  |  | *Wolbachia* (*w*A2 strain) | 1.00 | na | na | *Wolbachia w*A1 */ Arsenophonus* | 0.17 | na | na |
|  |  |  |  |  |  | *Arsenophonus* | 0.17 | na | na | *Wolbachia w*A2 */ Arsenophonus* | 0.17 | na | na |
|  |  |  |  |  |  |  |  |  |  | *Wolbachia w*A1 */ Wolbachia w*A2 / *Arsenophonus* | 0.17 | na | na |
|  | NEMATOCERA |  |  |  |  |  |  |  |  |  |  |  |  |
|  | **Culicidae** |  |  |  |  |  |  |  |  |  |  |  |  |
|  | *Aedes albopictus* | Roma, Italia, 2005 | 10 | 10 | - | *Wolbachia* (*w*AlbA strain) | 1.00 | 1.00 | 1.00 | *Wolbachia w*AlbA */ Wolbachia w*AlbB | 1.00 | 1.00 | 1.00 |
|  |  |  |  |  |  | *Wolbachia* (*w*AlbB strain) | 1.00 | 1.00 | 1.00 |  |  |  |  |
|  |  |  |  |  |  |  |  |  |  |  |  |  |  |
|  | **HEMIPTERA** |  |  |  |  |  |  |  |  |  |  |  |  |
|  | EUHEMIPTERA |  |  |  |  |  |  |  |  |  |  |  |  |
|  | **Cicadellidae** |  |  |  |  |  |  |  |  |  |  |  |  |
|  | *Cicadella viridis* | L'Olme, France, 2006 | 7 | 10 | - | *Wolbachia* | 0.94 | 0.86 | 1.00 | *Wolbachia / S. ixodetis* | 0.41 | 0.29 | 0.50 |
|  |  |  |  |  |  | *S. ixodetis* | 0.41 | 0.29 | 0.50 |  |  |  |  |
|  | **Miridae** |  |  |  |  |  |  |  |  |  |  |  |  |
|  | *Notostira elongata* | L'Olme, France, 2006 | - | - | 12 | *Wolbachia* | 1.00 | na | na | *Wolbachia / S. ixodetis* | 0.35 | na | na |
|  |  |  |  |  |  | *S. ixodetis* | 0.35 | na | na |  |  |  |  |
|  |  |  |  |  |  |  |  |  |  |  |  |  |  |
|  | **HYMENOPTERA** |  |  |  |  |  |  |  |  |  |  |  |  |
|  | ACULEATA |  |  |  |  |  |  |  |  |  |  |  |  |
|  | **Vespidae** |  |  |  |  |  |  |  |  |  |  |  |  |
|  | *Polistes nimpha* | St Nazaire de Pézan, France, 2006 | 10 | 10 | - | *Arsenophonus* (unnamed stain 1) | 1.00 | 1.00 | 1.00 | *Arsenophonus* (unnamed stain 1) */ Arsenophonus* (unnamed strain 2) | 1.00 | 1.00 | 1.00 |
|  |  |  |  |  |  | *Arsenophonus* (unnamed stain 2) | 1.00 | 1.00 | 1.00 |  |  |  |  |
|  |  |  |  |  |  |  |  |  |  |  |  |  |  |

**Table S2.** Results of arthropod screening for species with coinfection, detailing frequency of coinfection. Only *M. mengei* displayed a significant difference between sexes in *Wolbachia* prevalence after a Bonferroni correction. na, not ascertained; ov., overall; un., undetermined.
