# Supplementary material for: The diversity of reproductive parasites among arthropods: Wolbachia do not walk alone
Source: BMC Biol. 2008 Jun 24;6:27. doi: 10.1186/1741-7007-6-27 (PMC2492848; doi:10.1186/1741-7007-6-27)
Supplement: Additional file 3 — Wolbachia 16S rDNA (A) and wsp (B) phylogenies constructed via neighbour-joining as implemented on MEGA version 3.1. The symbionts have the prefix S followed by the proper name of their host. Sequences from this study are underlined and some previously published Wolbachia sequences are shown in plain type. Effect of infection is indicated in bold type if known (CI, cytoplasmic incompatibility; P, parthenogenesis; F, feminisation). Major Wolbachia supergroup lineages are reported (A)-(H). Numbers on branches indicate percentage bootstrap support for major branches (1000 replicates; only bootstrap values of 60% or more are shown). [file 1741-7007-6-27-S3.doc]

A

B
