# Supplementary material for: The diversity of reproductive parasites among arthropods: Wolbachia do not walk alone
Source: BMC Biol. 2008 Jun 24;6:27. doi: 10.1186/1741-7007-6-27 (PMC2492848; doi:10.1186/1741-7007-6-27)
Supplement: Supplementary file 4 [file 1741-7007-6-27-S4.doc]

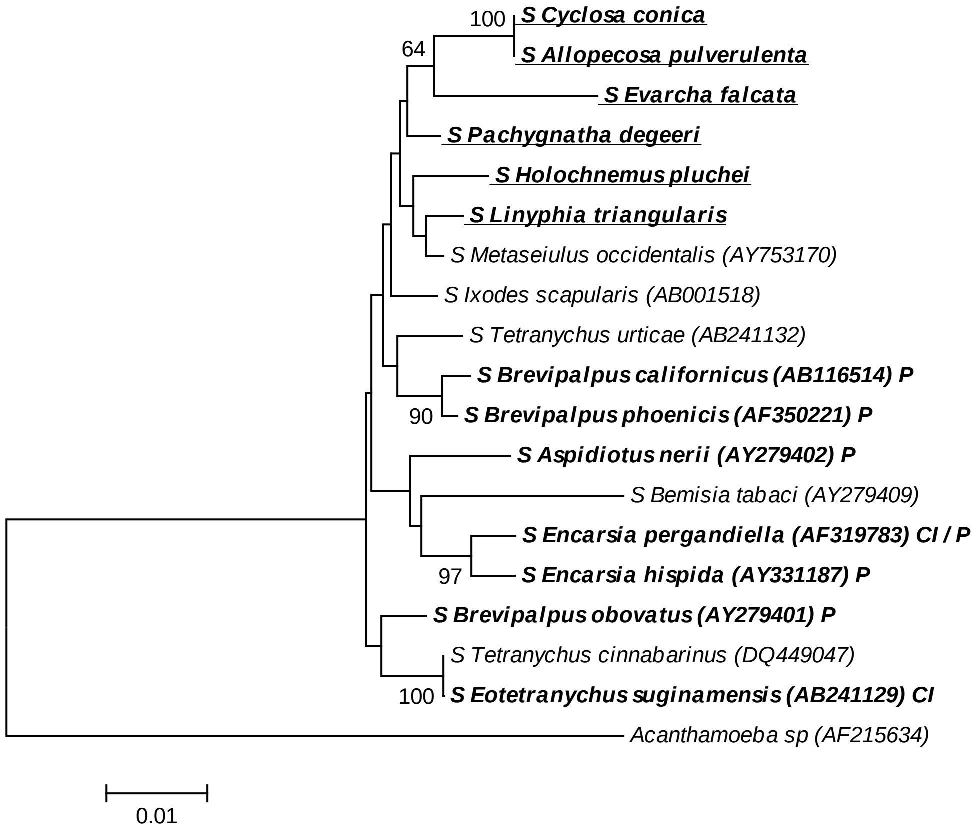
100
64
S Cyclosa conica
S Allopecosa pulverulenta
S Evarcha falcata
S Pachygnatha degeeri
S Holochnemus pluchei
S Linyphia triangularis
S Metaseiulus occidentalis (AY753170)
S Ixodes scapularis (AB001518)
S Tetranychus urticae (AB241132)
S Brevipalpus californicus (AB116514) P
90
S Brevipalpus phoenicis (AF350221) P
S Aspidiotus nerii (AY279402) P
S Bemisia tabaci (AY279409)
S Encarsia pergandiella (AF319783) CI / P
97
S Encarsia hispida (AY331187) P
S Brevipalpus obovatus (AY279401) P
S Tetranychus cinnabarinus (DQ449047)
100
S Eotetranychus suginamensis (AB241129) CI
Acanthamoeba sp (AF215634)
0.01
