# Supplementary material for: The diversity of reproductive parasites among arthropods: Wolbachia do not walk alone
Source: BMC Biol. 2008 Jun 24;6:27. doi: 10.1186/1741-7007-6-27 (PMC2492848; doi:10.1186/1741-7007-6-27)
Supplement: Supplementary file 5 [file 1741-7007-6-27-S5.doc]

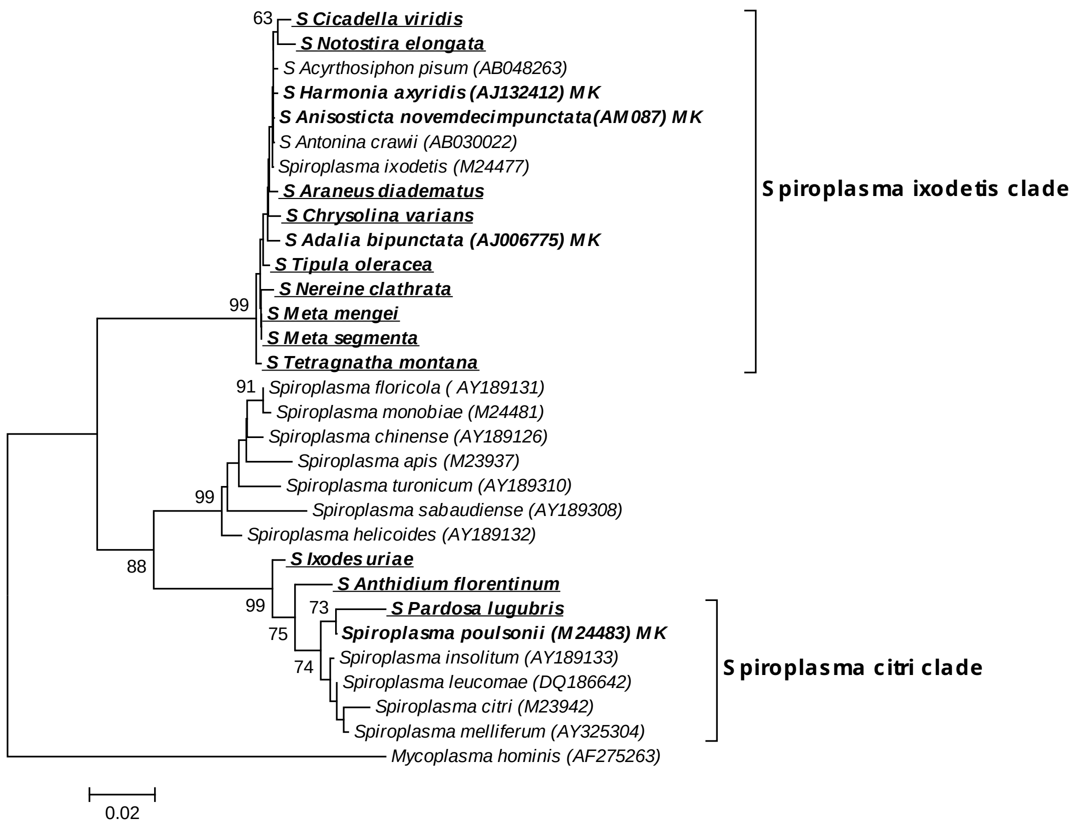

63
S Cicadella viridis
S Notostira elongata
S Acyrthosiphon pisum (AB048263)
S Harmonia axyridis (AJ132412) MK
S Anisosticta novemdecimpunctata(AM087) MK
S Antonina crawii (AB030022)
Spiroplasma ixodetis (M24477)
S Araneus diadematus
S Chrysolina varians
S Adalia bipunctata (AJ006775) MK
S Tipula oleracea
S Nereine clathrata
99
S Meta mengei
S Meta segmenta
S Tetragnatha montana
Spiroplasma ixodetis clade
91
Spiroplasma floricola (AY189131)
Spiroplasma monobiae (M24481)
Spiroplasma chinense (AY189126)
Spiroplasma apis (M23937)
Spiroplasma turonicum (AY189310)
99
Spiroplasma sabaudiense (AY189308)
Spiroplasma helicoides (AY189132)
S Ixodes uriae
88
S Anthidium florentinum
99
73
S Pardosa lugubris
75
Spiroplasma poulsonii (M24483) MK
74
Spiroplasma insolitum (AY189133)
Spiroplasma leucomae (DQ186642)
Spiroplasma citri (M23942)
Spiroplasma melliferum (AY325304)
Spiroplasma citri clade
Mycoplasma hominis (AF275263)
0.02
