# Supplementary material for: The diversity of reproductive parasites among arthropods: Wolbachia do not walk alone
Source: BMC Biol. 2008 Jun 24;6:27. doi: 10.1186/1741-7007-6-27 (PMC2492848; doi:10.1186/1741-7007-6-27)
Supplement: Supplementary file 6 [file 1741-7007-6-27-S6.doc]

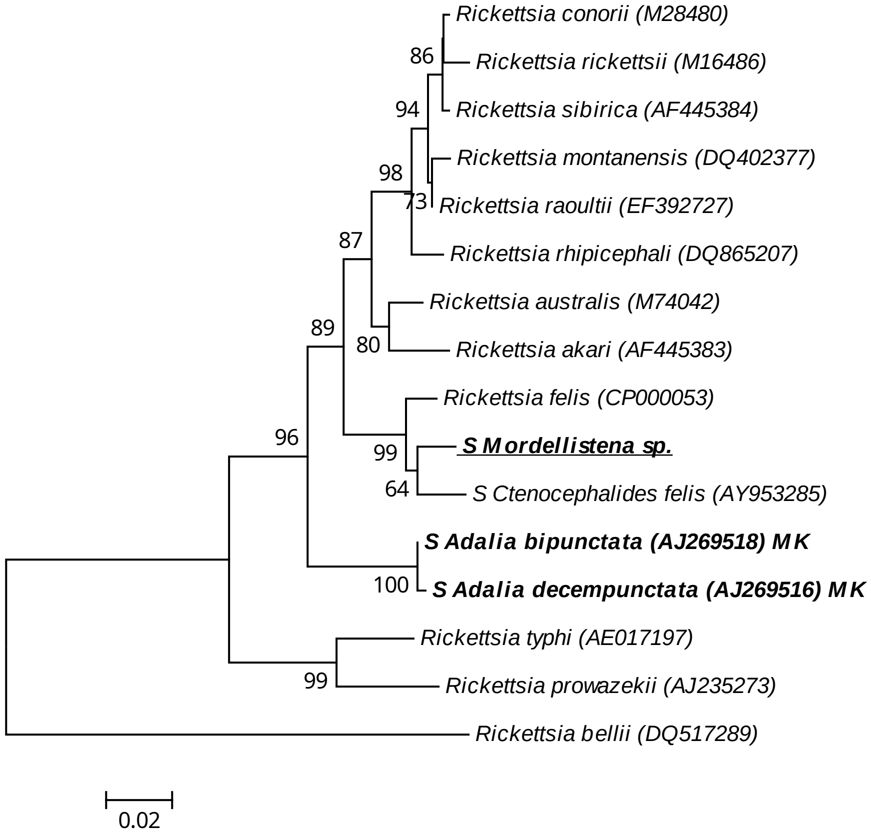
Rickettsia conorii (M28480)
Rickettsia rickettsii (M16486)
Rickettsia sibirica (AF445384)
Rickettsia montanensis (DQ402377)
Rickettsia raoultii (EF392727)
Rickettsia rhipicephali (DQ865207)
Rickettsia australis (M74042)
Rickettsia akari (AF445383)
Rickettsia felis (CP000053)
S Mordellistena sp.
S Ctenocephalides felis (AY953285)
S Adalia bipunctata (AJ269518) MK
S Adalia decempunctata (AJ269516) MK
Rickettsia typhi (AE017197)
Rickettsia prowazekii (AJ235273)
Rickettsia bellii (DQ517289)
86
94
98
73
87
89
80
96
99
64
100
99
0.02
