# Supplementary material for: The diversity of reproductive parasites among arthropods: Wolbachia do not walk alone
Source: BMC Biol. 2008 Jun 24;6:27. doi: 10.1186/1741-7007-6-27 (PMC2492848; doi:10.1186/1741-7007-6-27)
Supplement: Supplementary file 7 [file 1741-7007-6-27-S7.doc]

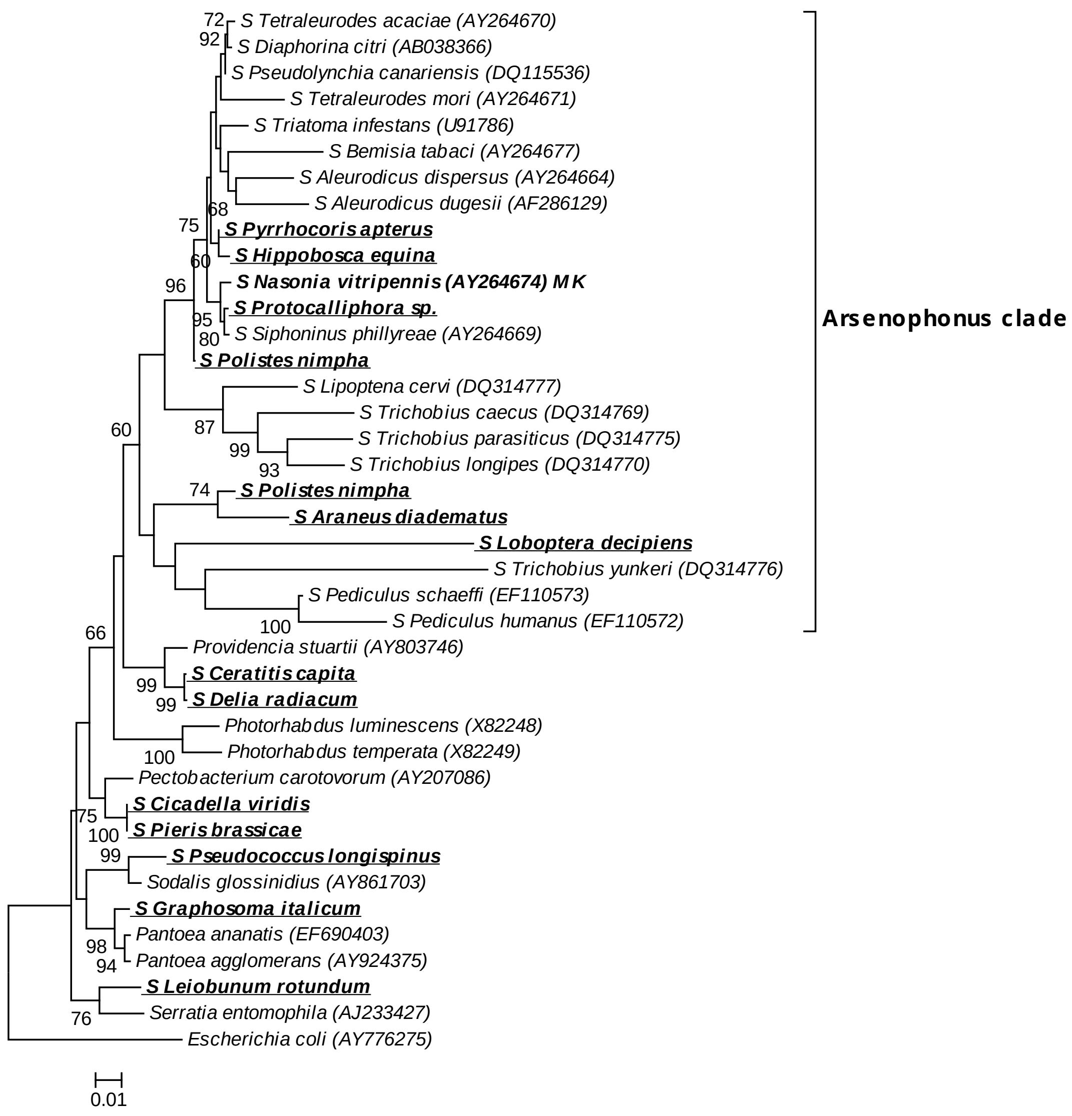
S Tetraleurodes acaciae (AY264670)
S Diaphorina citri (AB038366)
S Pseudolynchia canariensis (DQ115536)
S Tetraleurodes mori (AY264671)
S Triatoma infestans (U91786)
S Bemisia tabaci (AY264677)
S Aleurodicus dispersus (AY264664)
S Aleurodicus dugesii (AF286129)
S Pyrrhocoris apterus
S Hippobosca equina
S Nasonia vitripennis (AY264674) MK
S Protocalliphora sp.
S Siphoninus phillyreae (AY264669)
S Polistes nimpha
S Lipoptena cervi (DQ314777)
S Trichobius caecus (DQ314769)
S Trichobius parasiticus (DQ314775)
S Trichobius longipes (DQ314770)
S Polistes nimpha
S Araneus diadematus
S Loboptera decipiens
S Trichobius yunkeri (DQ314776)
S Pediculus schaeffi (EF110573)
S Pediculus humanus (EF110572)
Arsenophonus clade
Providencia stuartii (AY803746)
S Ceratitis capita
S Delia radiacum
Photorhabdus luminescens (X82248)
Photorhabdus temperata (X82249)
Pectobacterium carotovorum (AY207086)
S Cicadella viridis
S Pieris brassicae
S Pseudococcus longispinus
Sodalis glossinidius (AY861703)
S Graphosoma italicum
Pantoea ananatis (EF690403)
Pantoea agglomerans (AY924375)
S Leiobunum rotundum
Serratia entomophila (AJ233427)
Escherichia coli (AY776275)
72
92
68
75
60
96
95
80
87
99
93
60
74
100
66
99
99
100
75
100
99
98
94
76
0.01
